# Supplementary figures and images for: NANOG governs cell metabolism and redox homeostasis in human naïve embryonic stem cells
Source: EMBO Rep. 2025 Nov 19;26(24):6292–324. doi: 10.1038/s44319-025-00629-9 (PMC12714712; doi:10.1038/s44319-025-00629-9)

Figure 1A

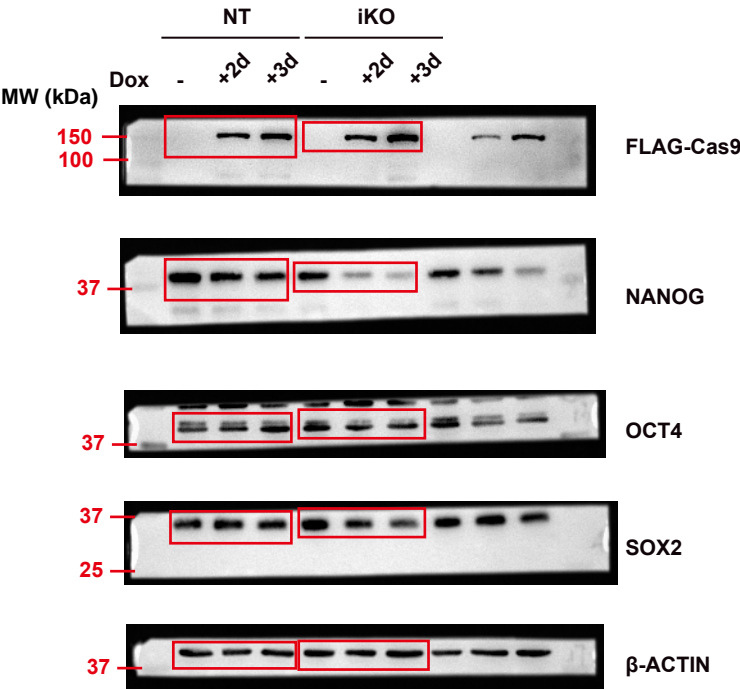

Supplement: Supplementary file 2 — Source data Fig. 1 [file 44319_2025_629_MOESM2_ESM.zip › Figure 1/1A/Fig 1A image.pdf]

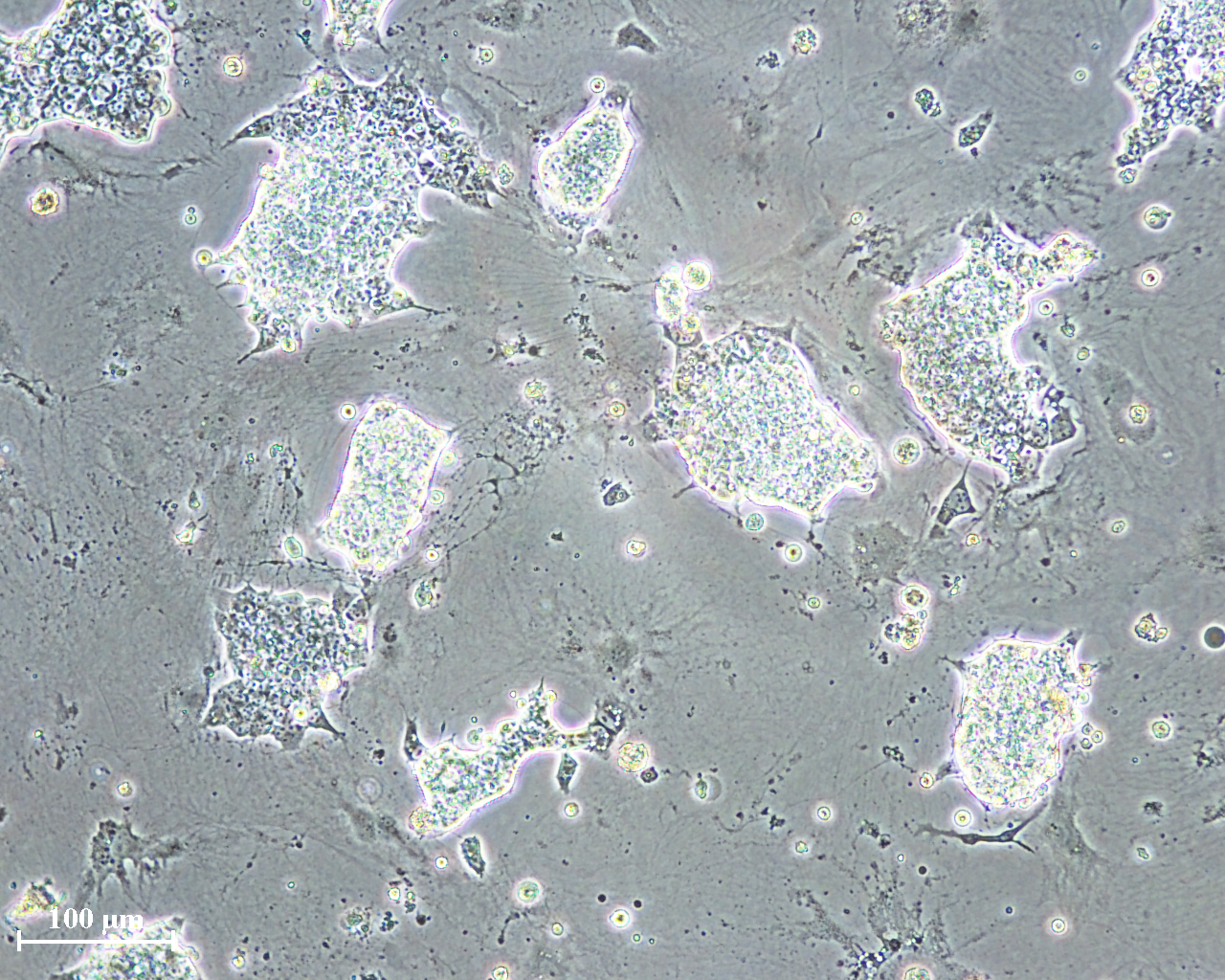

Supplement: Supplementary file 2 — Source data Fig. 1 [file 44319_2025_629_MOESM2_ESM.zip › Figure 1/1B/NT/NT +DOX 2d 10X.tif]

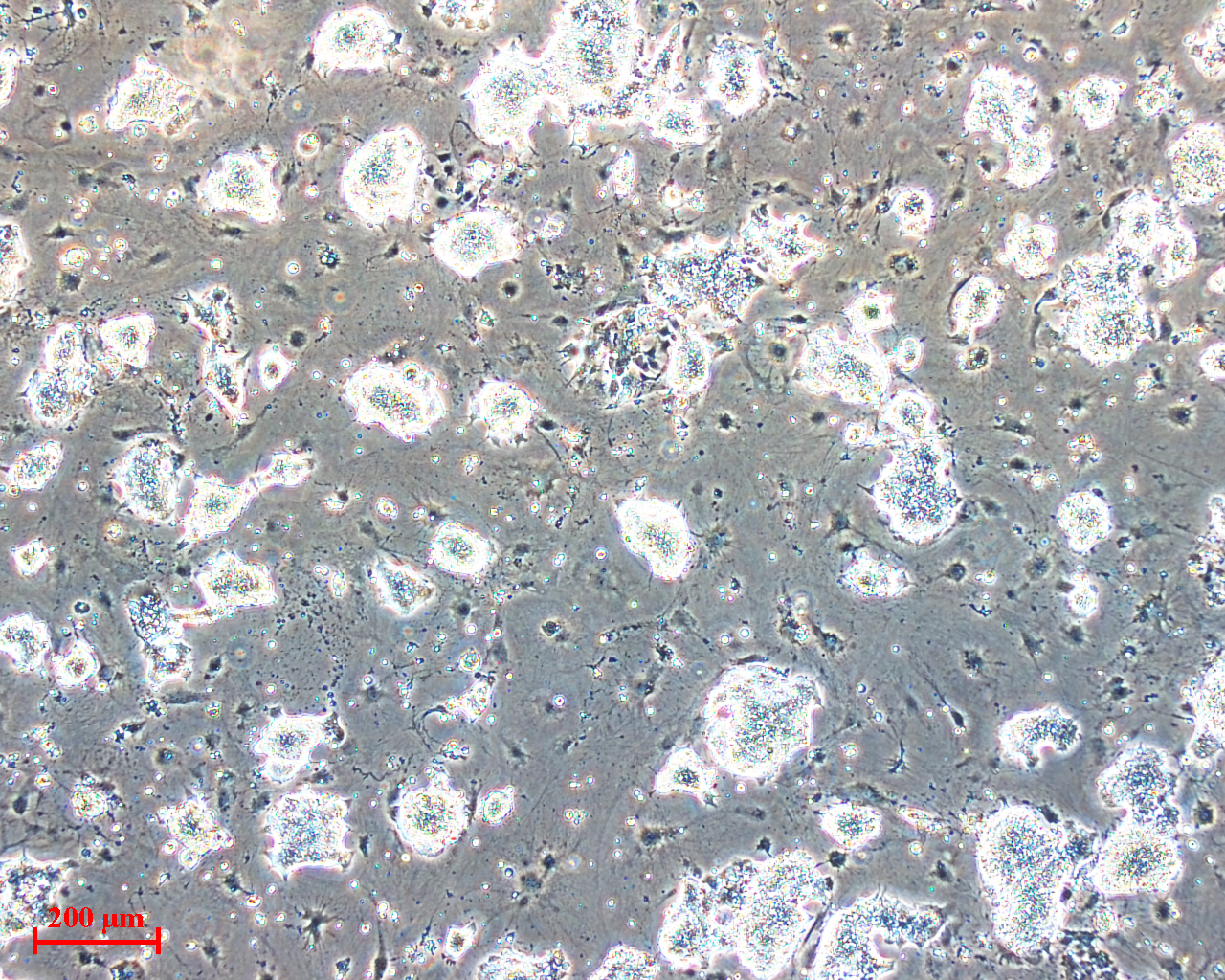

Supplement: Supplementary file 2 — Source data Fig. 1 [file 44319_2025_629_MOESM2_ESM.zip › Figure 1/1B/NT/NT +DOX 2d 4X.tif]

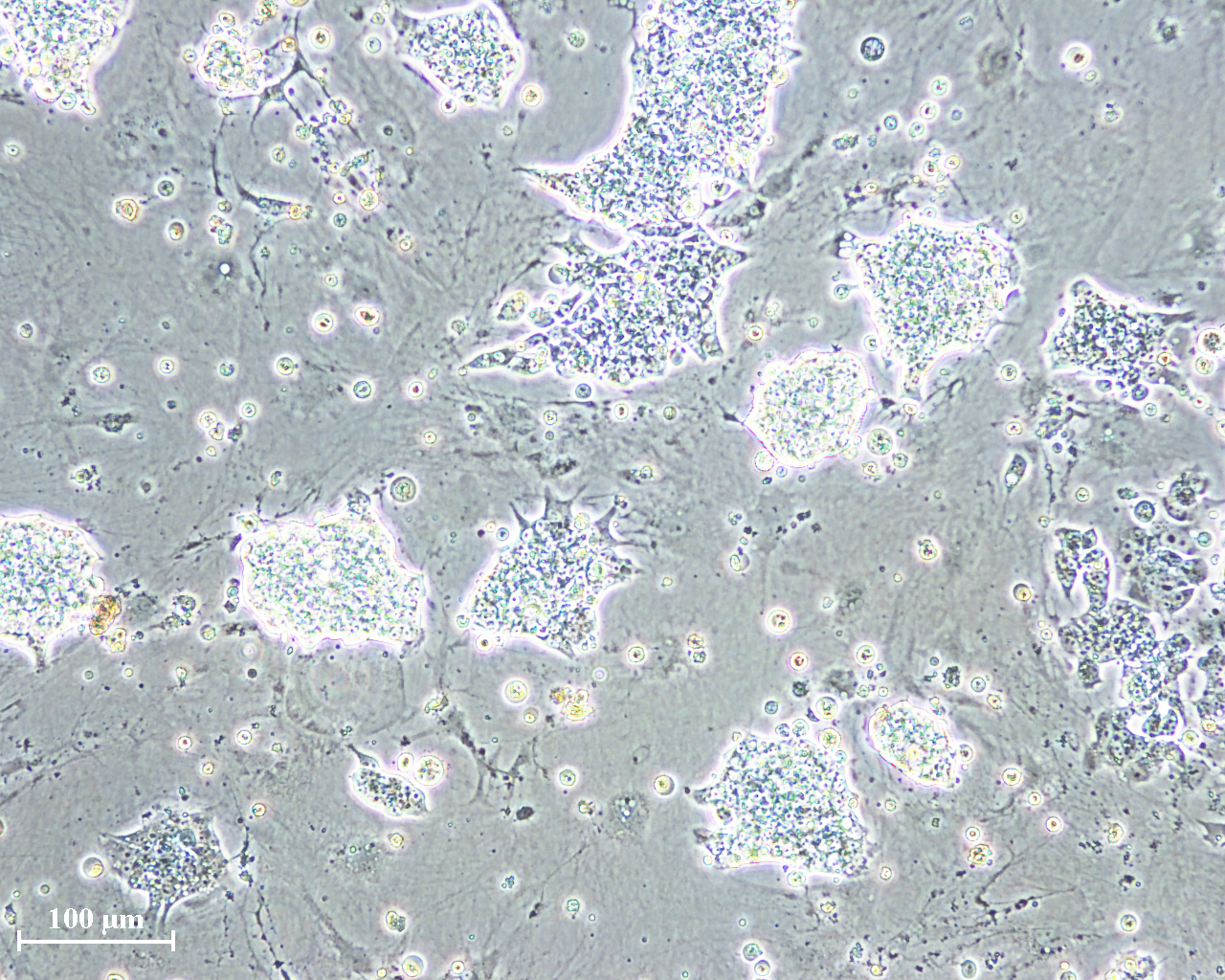

Supplement: Supplementary file 2 — Source data Fig. 1 [file 44319_2025_629_MOESM2_ESM.zip › Figure 1/1B/NT/NT +DOX 3d 10X.tif]

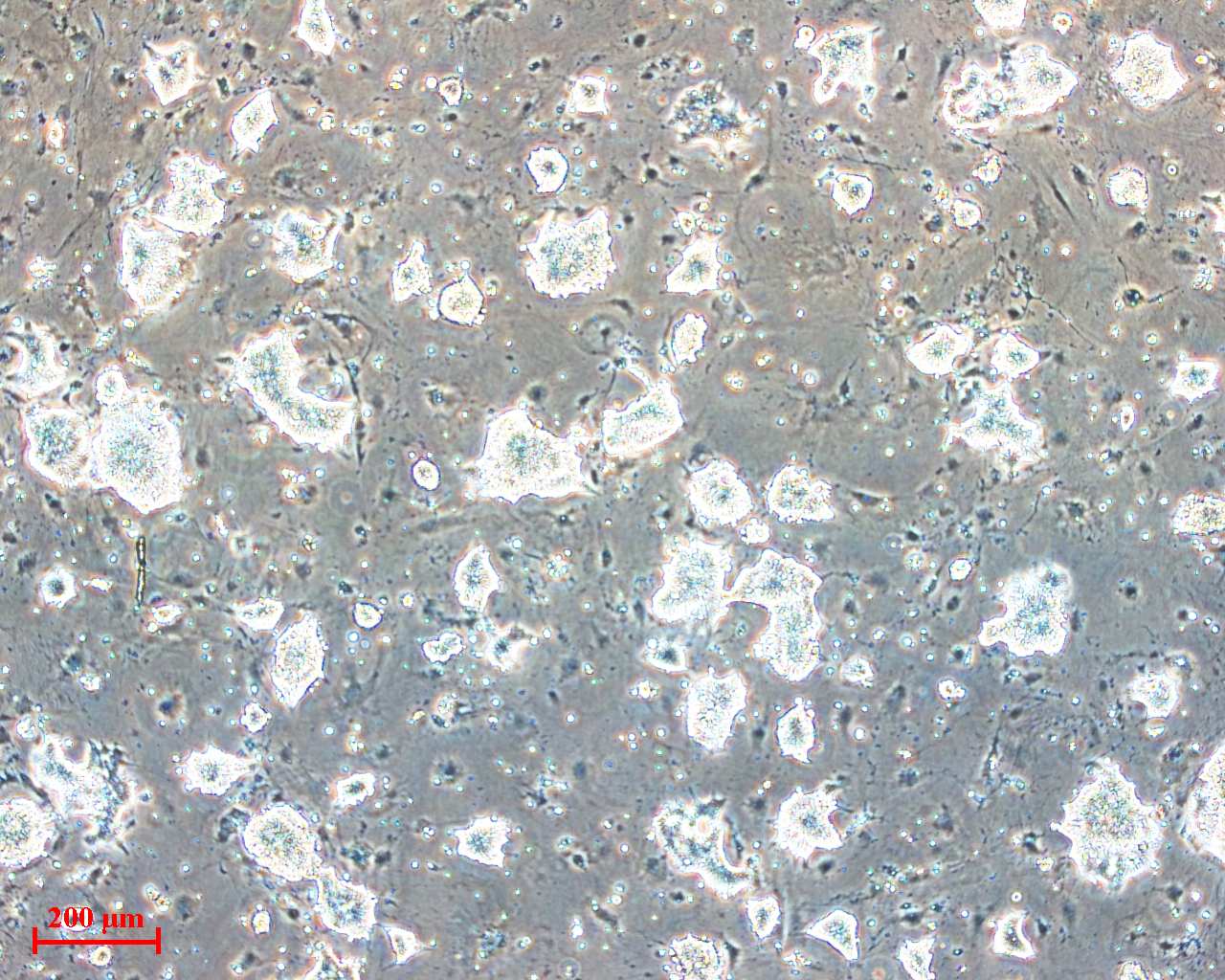

Supplement: Supplementary file 2 — Source data Fig. 1 [file 44319_2025_629_MOESM2_ESM.zip › Figure 1/1B/NT/NT +DOX 3d 4X.tif]

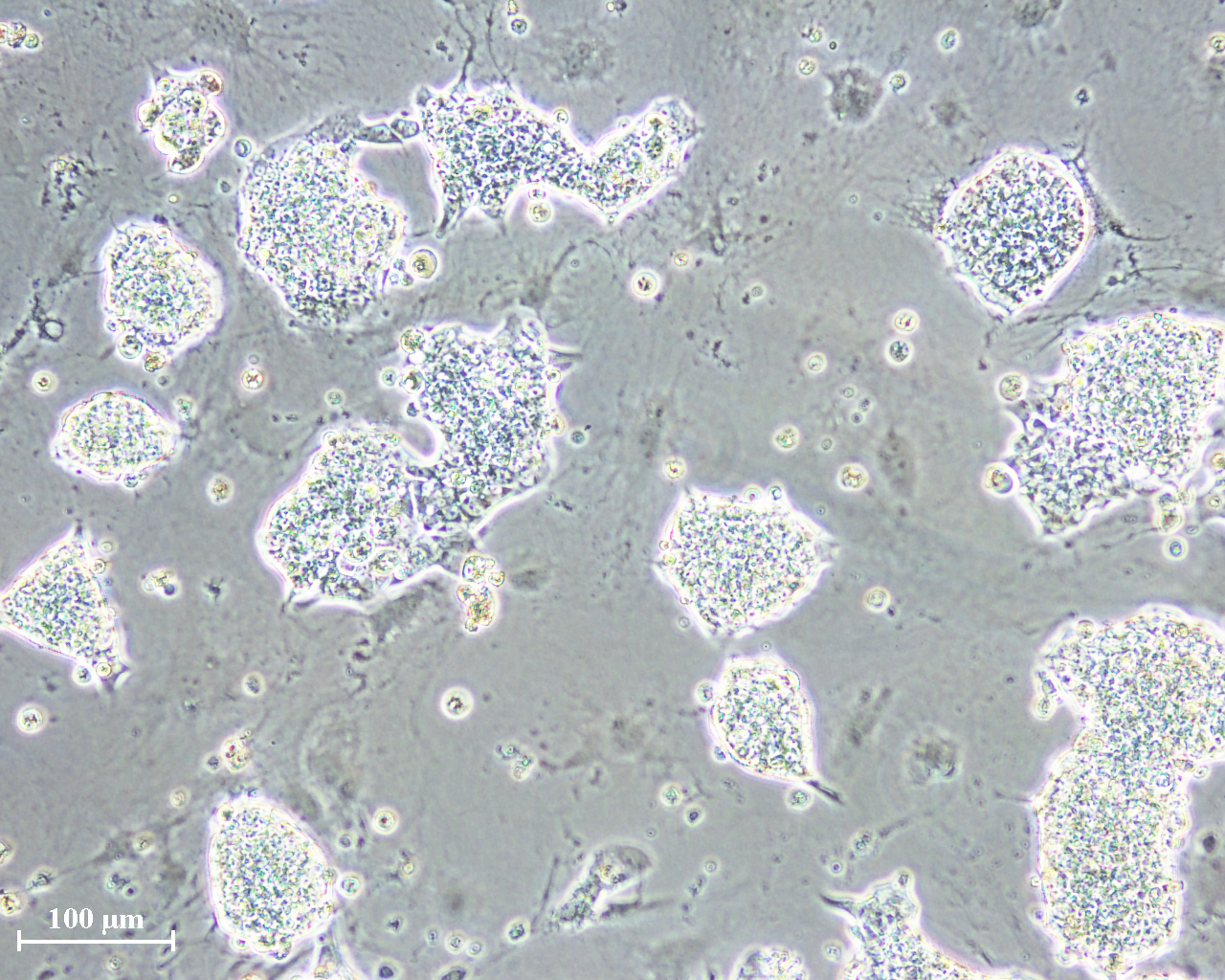

Supplement: Supplementary file 2 — Source data Fig. 1 [file 44319_2025_629_MOESM2_ESM.zip › Figure 1/1B/NT/NT -DOX 10X.tif]

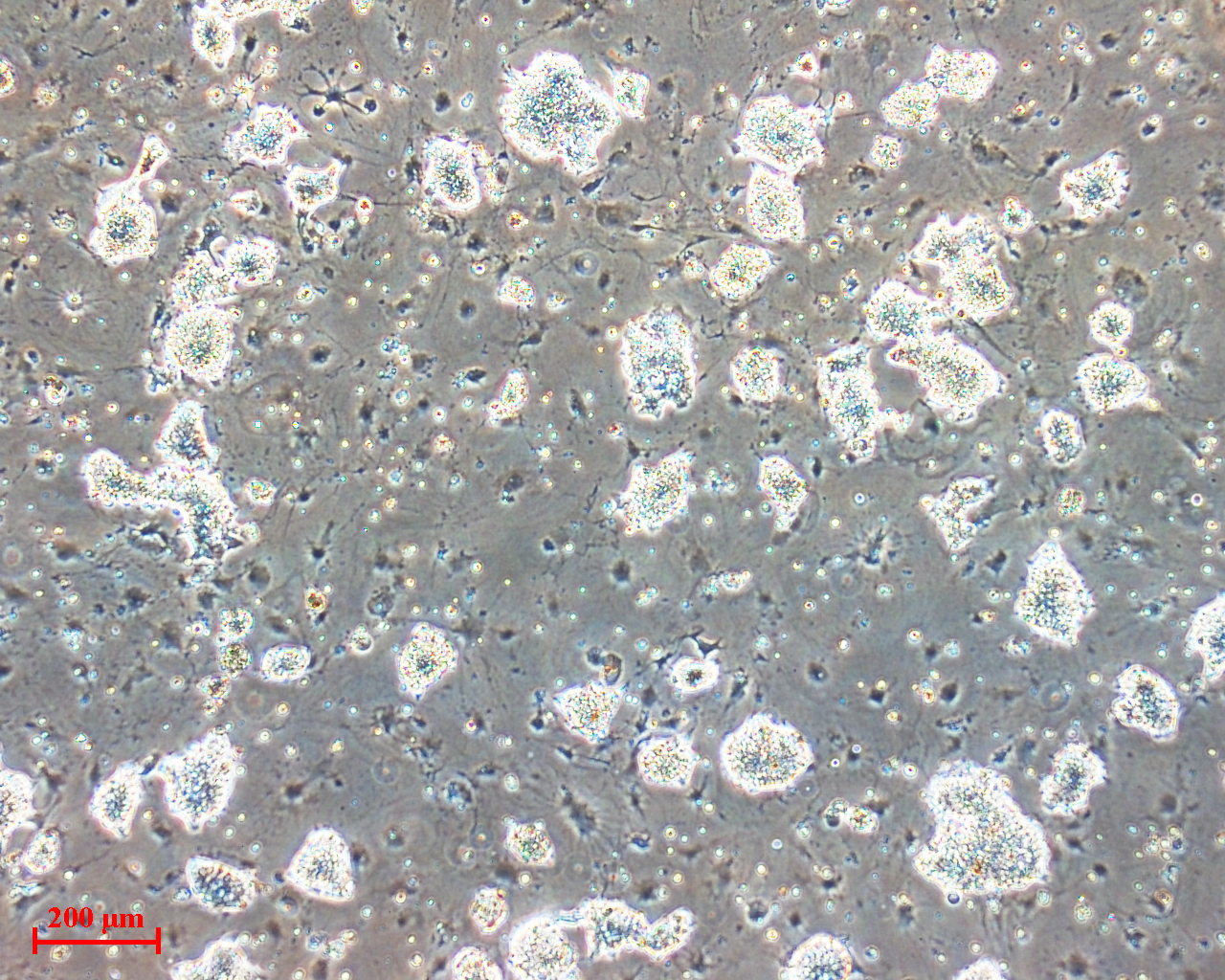

Supplement: Supplementary file 2 — Source data Fig. 1 [file 44319_2025_629_MOESM2_ESM.zip › Figure 1/1B/NT/NT -DOX 4X.tif]

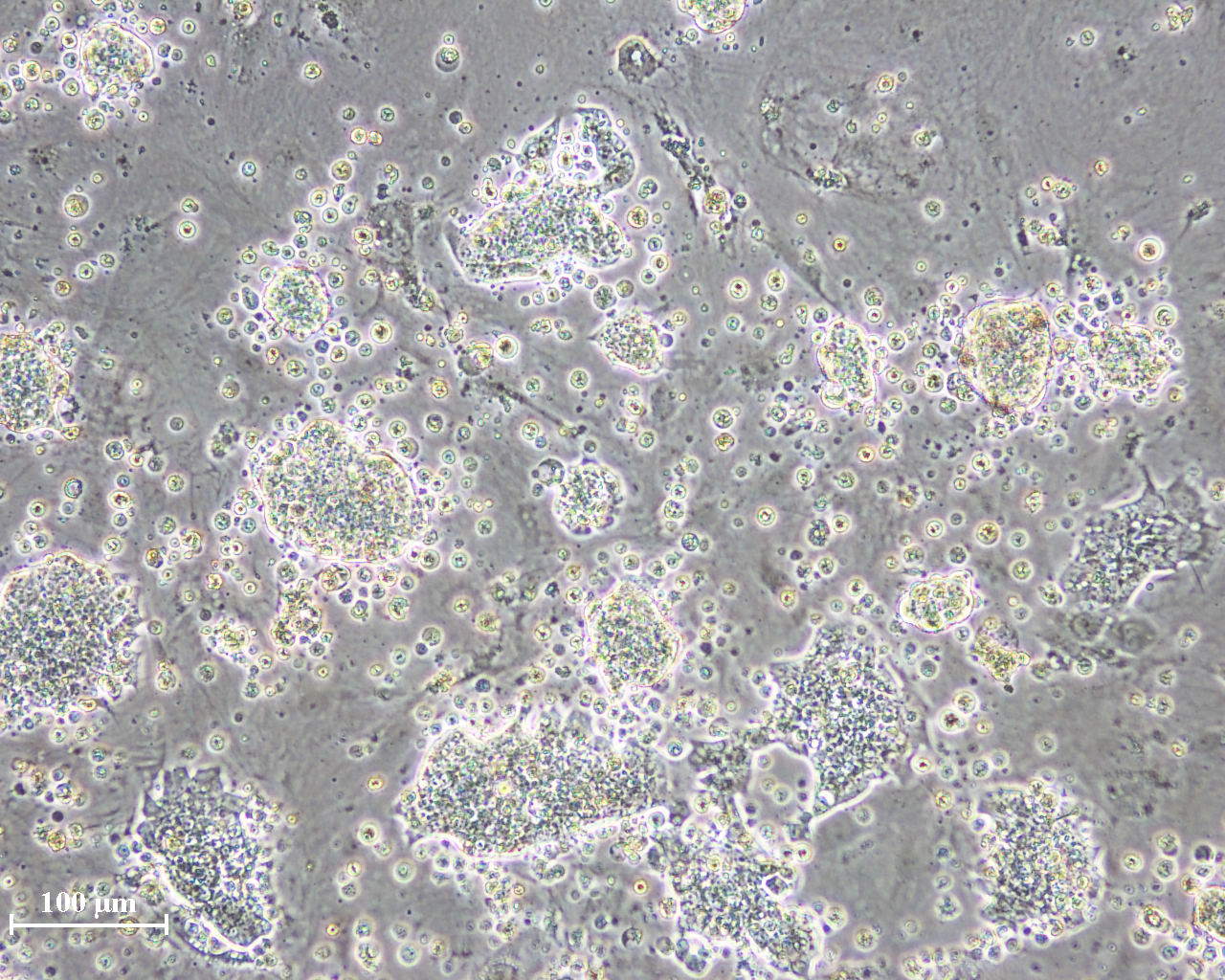

Supplement: Supplementary file 2 — Source data Fig. 1 [file 44319_2025_629_MOESM2_ESM.zip › Figure 1/1B/iKO/iKO +DOX 2d 10x.tif]

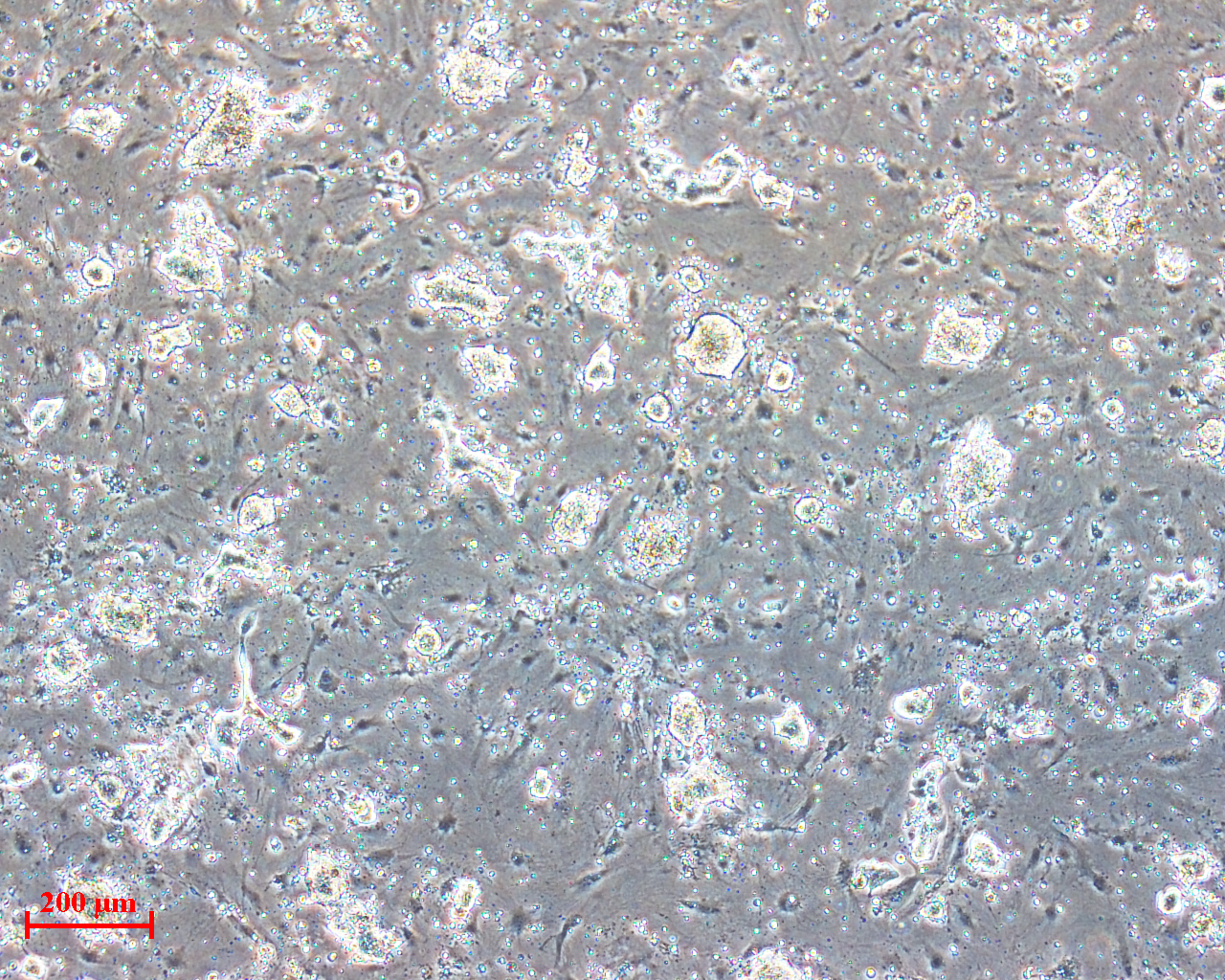

Supplement: Supplementary file 2 — Source data Fig. 1 [file 44319_2025_629_MOESM2_ESM.zip › Figure 1/1B/iKO/iKO +DOX 2d 4x.tif]

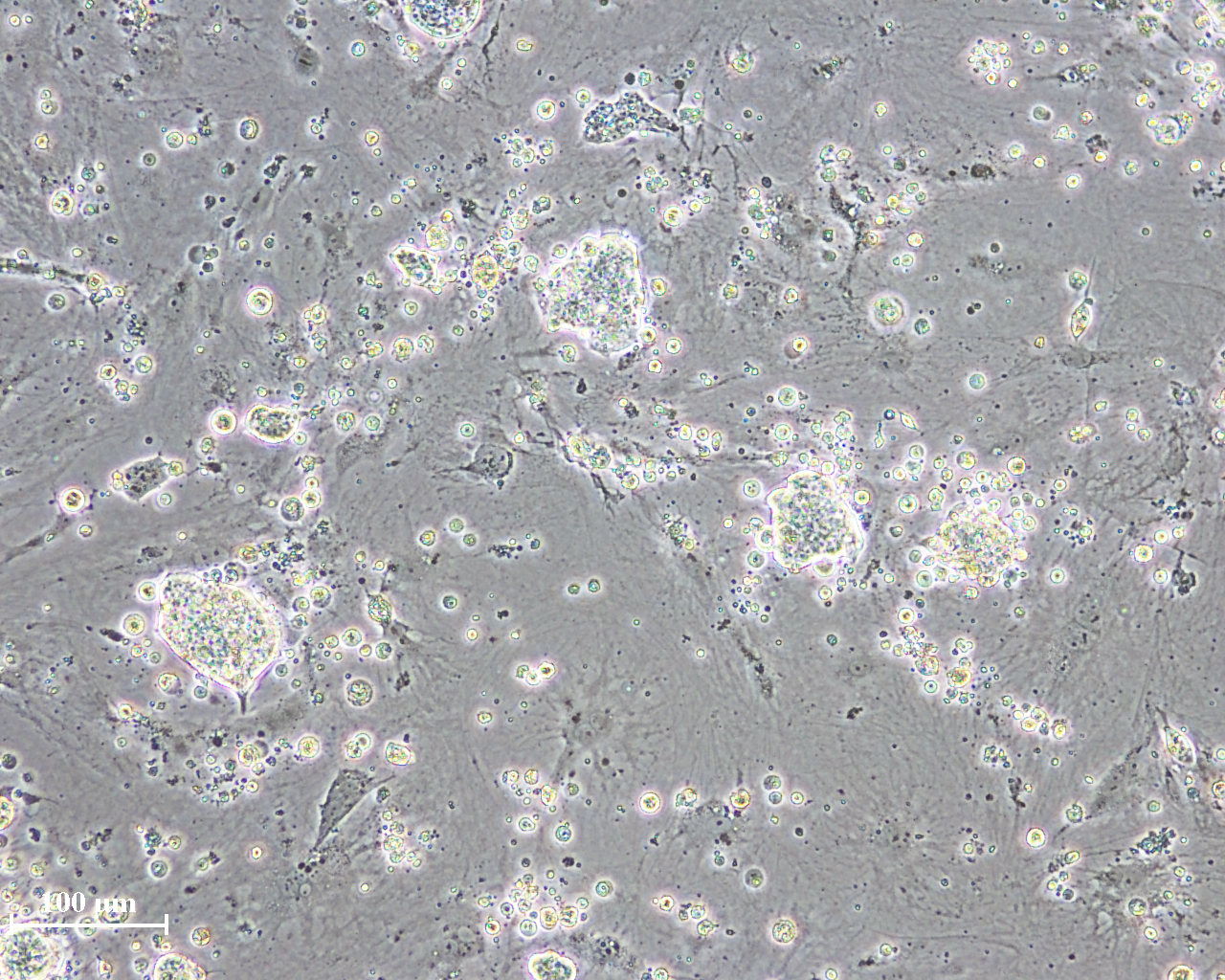

Supplement: Supplementary file 2 — Source data Fig. 1 [file 44319_2025_629_MOESM2_ESM.zip › Figure 1/1B/iKO/iKO +DOX 3d 10x.tif]

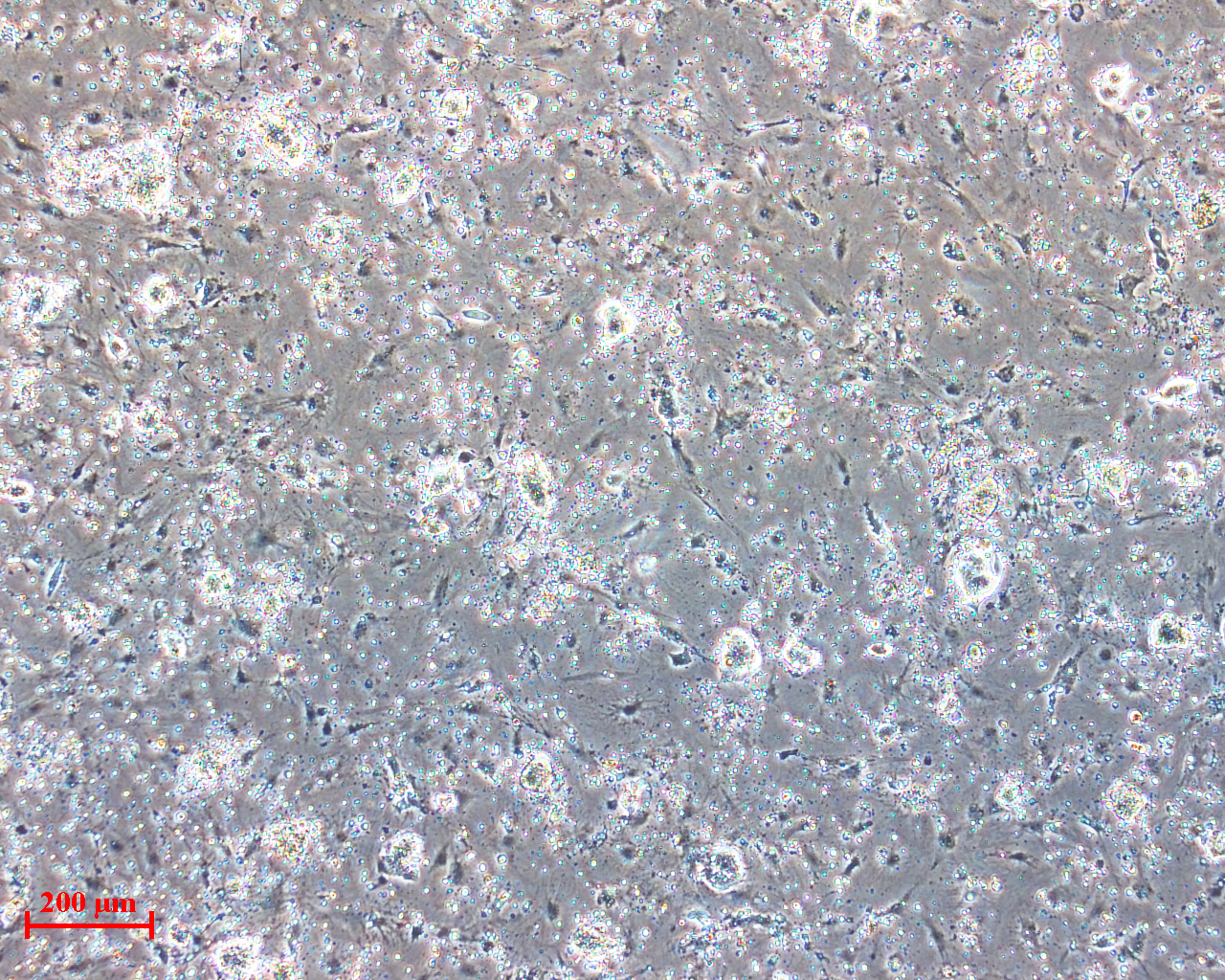

Supplement: Supplementary file 2 — Source data Fig. 1 [file 44319_2025_629_MOESM2_ESM.zip › Figure 1/1B/iKO/iKO +DOX 3d 4x.tif]

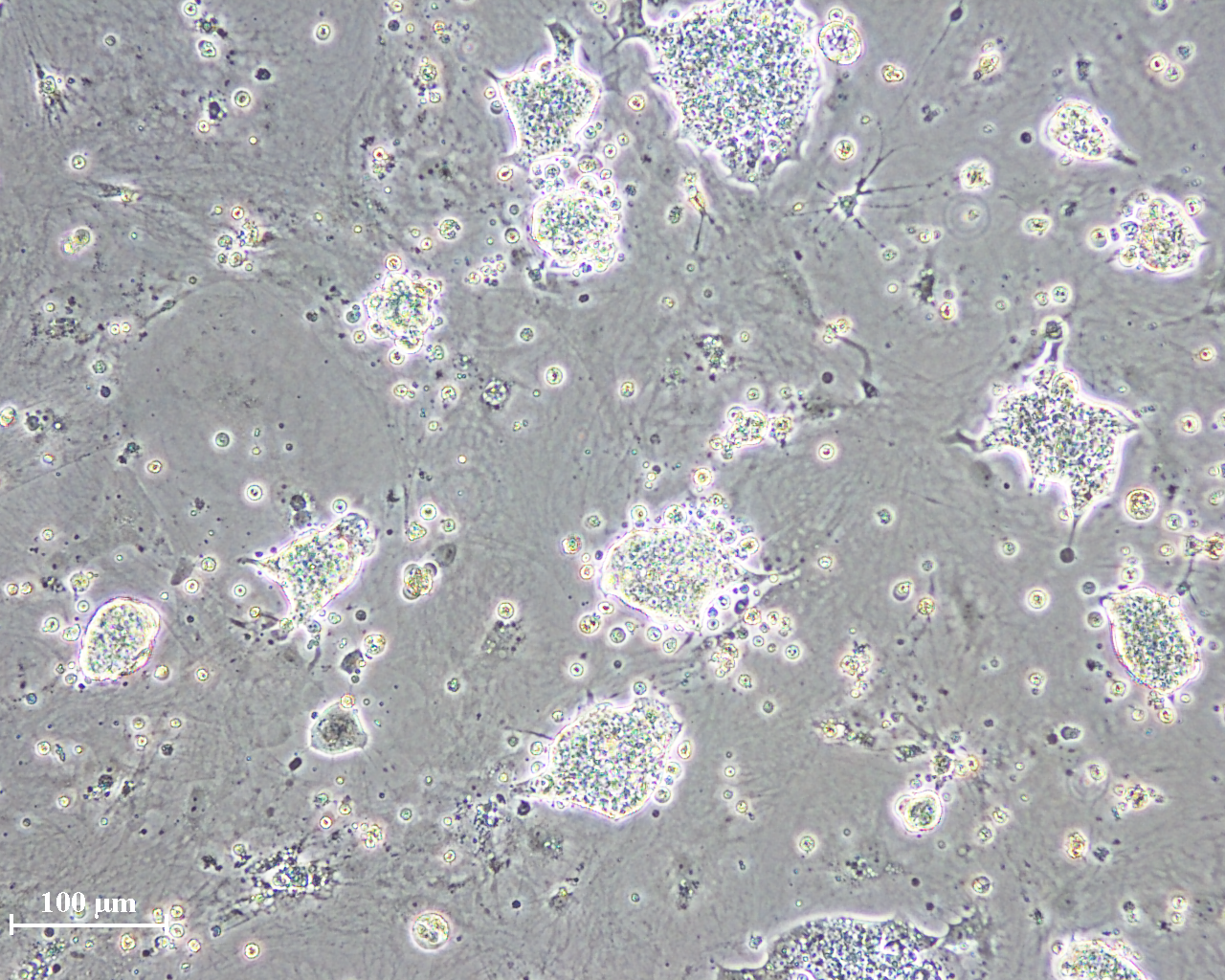

Supplement: Supplementary file 2 — Source data Fig. 1 [file 44319_2025_629_MOESM2_ESM.zip › Figure 1/1B/iKO/iKO -DOX 10X.tif]

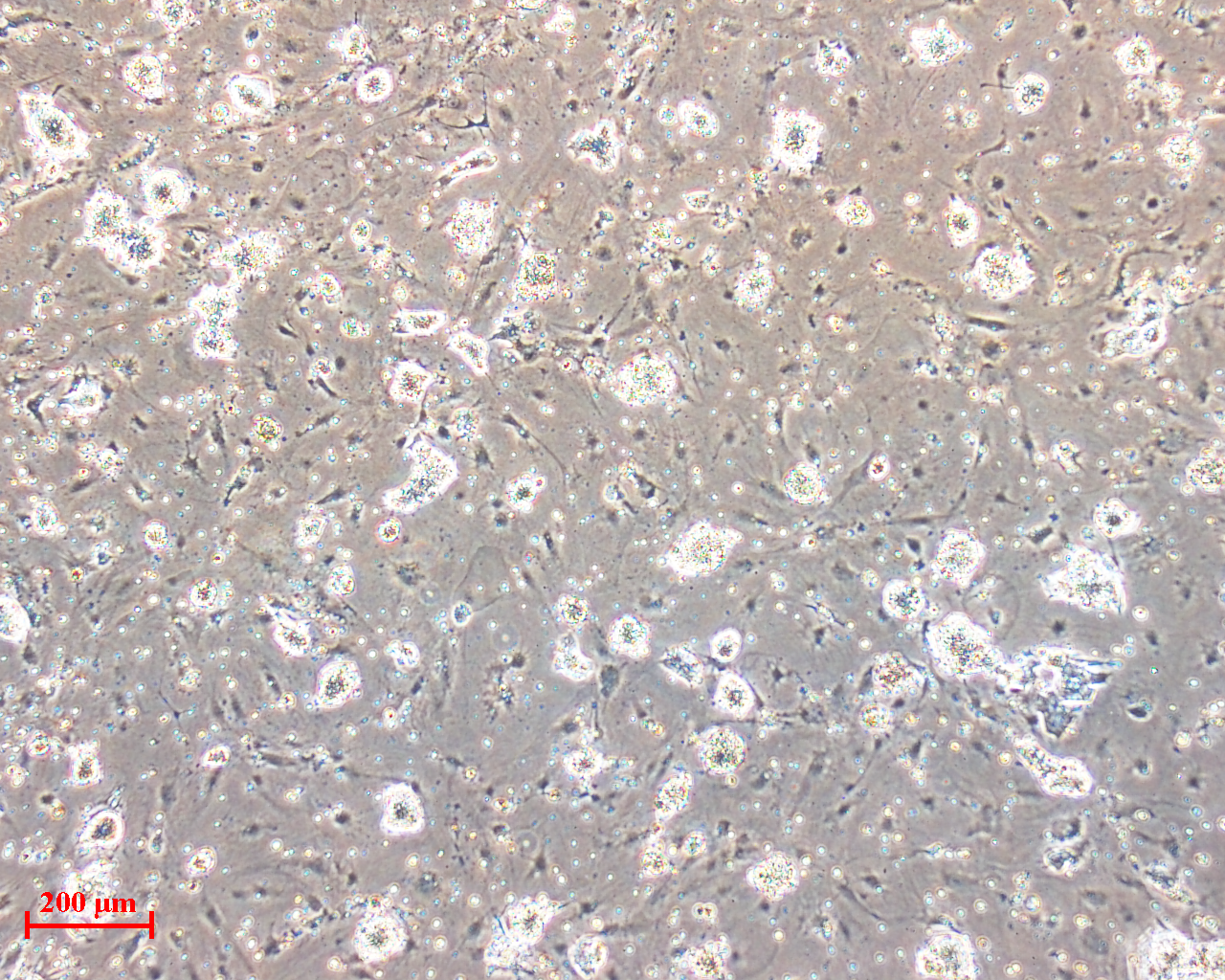

Supplement: Supplementary file 2 — Source data Fig. 1 [file 44319_2025_629_MOESM2_ESM.zip › Figure 1/1B/iKO/iKO -DOX 4X.tif]

Figure 1F

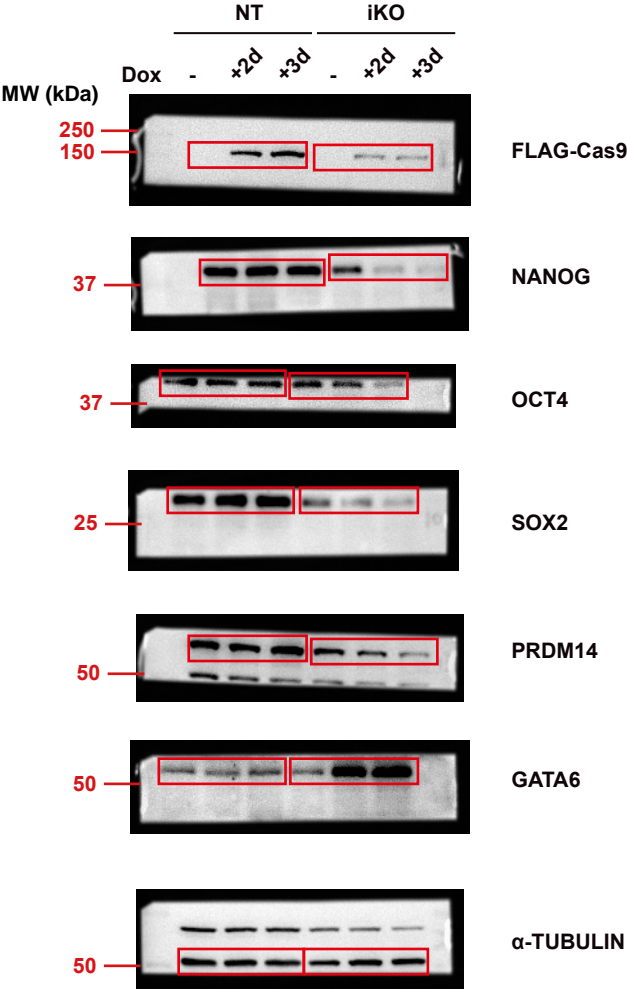

Supplement: Supplementary file 2 — Source data Fig. 1 [file 44319_2025_629_MOESM2_ESM.zip › Figure 1/1F/Fig 1F image.pdf]

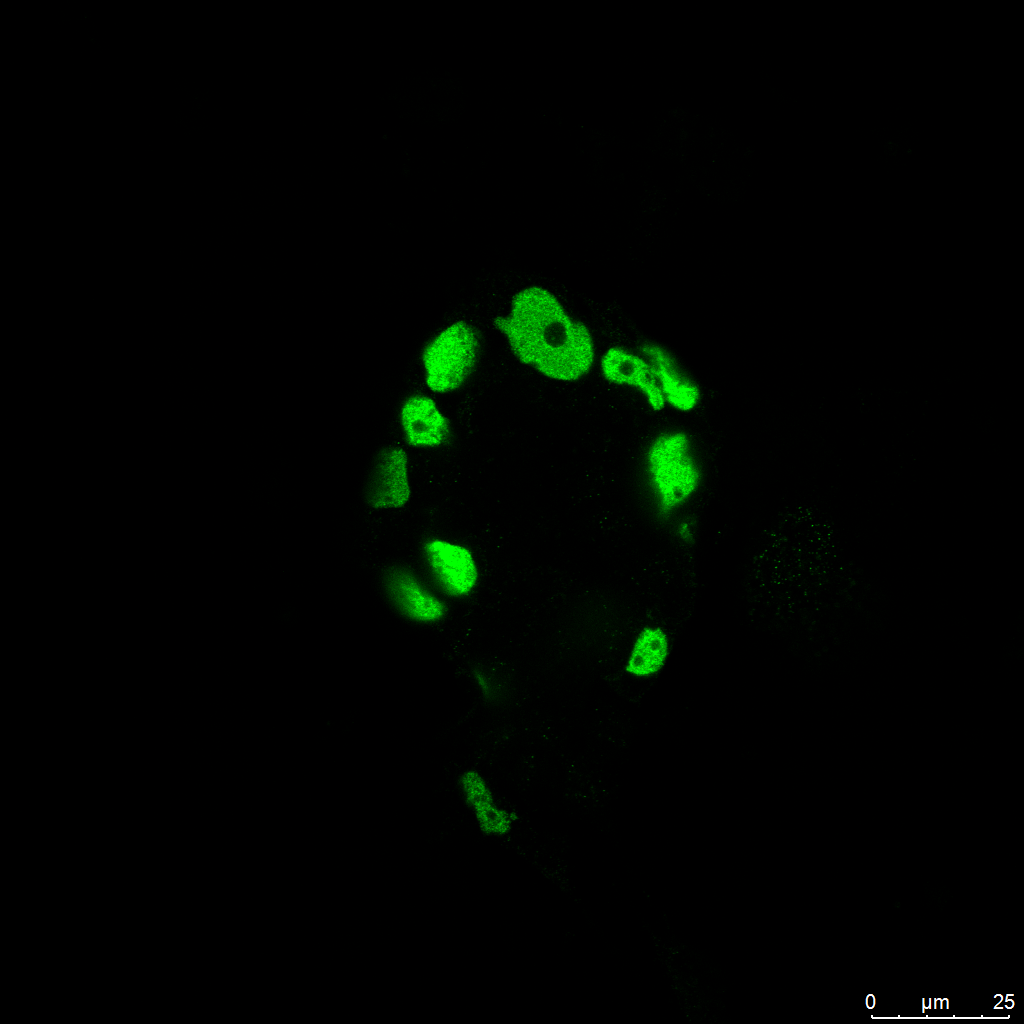

Supplement: Supplementary file 2 — Source data Fig. 1 [file 44319_2025_629_MOESM2_ESM.zip › Figure 1/1H/Left/+DOX 2d GATA3.tif]

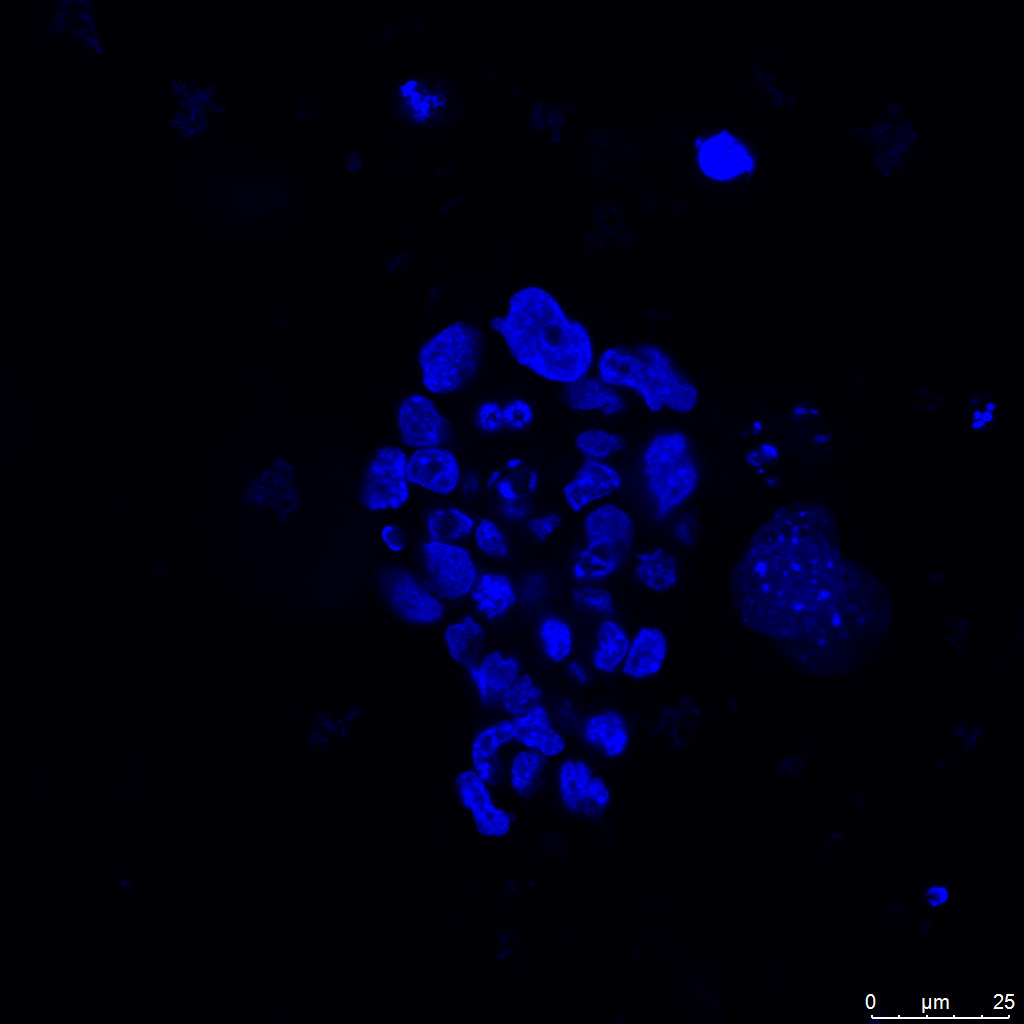

Supplement: Supplementary file 2 — Source data Fig. 1 [file 44319_2025_629_MOESM2_ESM.zip › Figure 1/1H/Left/+DOX 2d Hoechst.tif]

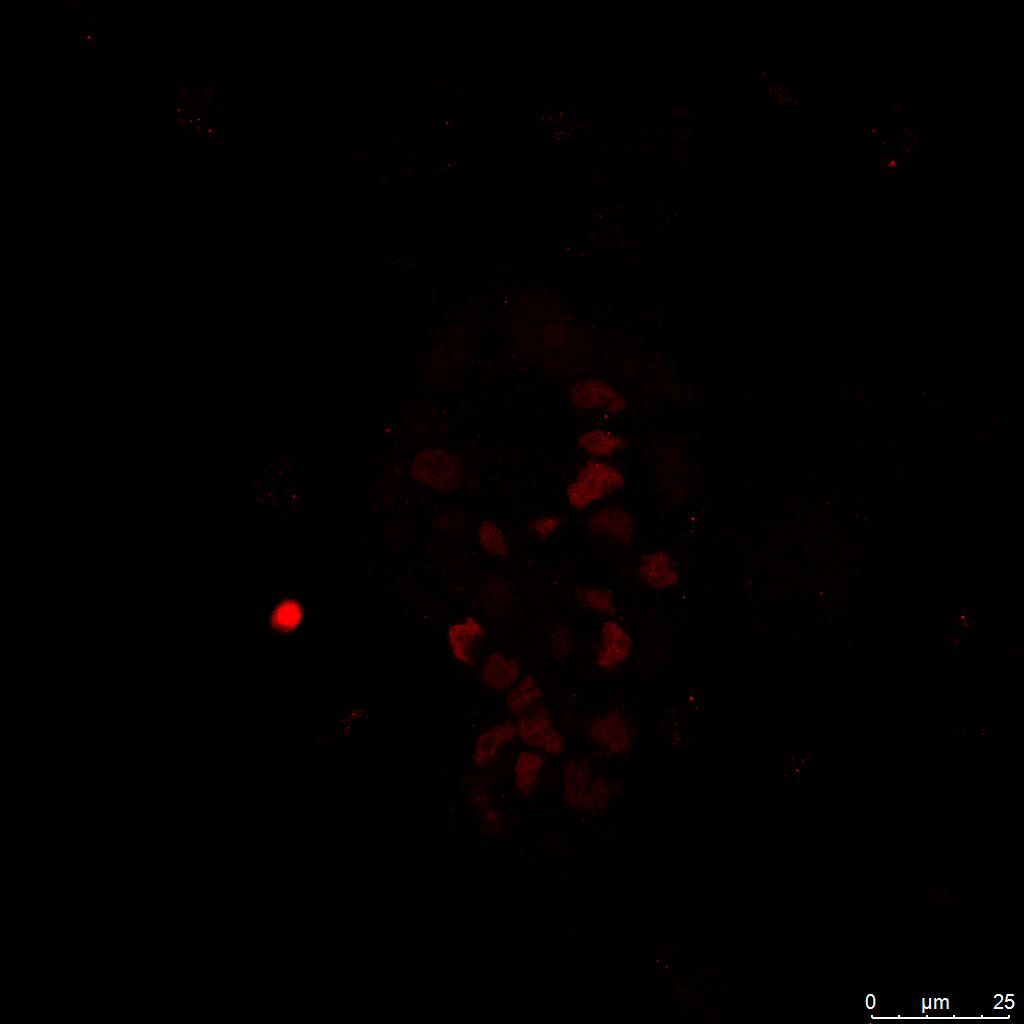

Supplement: Supplementary file 2 — Source data Fig. 1 [file 44319_2025_629_MOESM2_ESM.zip › Figure 1/1H/Left/+DOX 2d NANOG.tif]

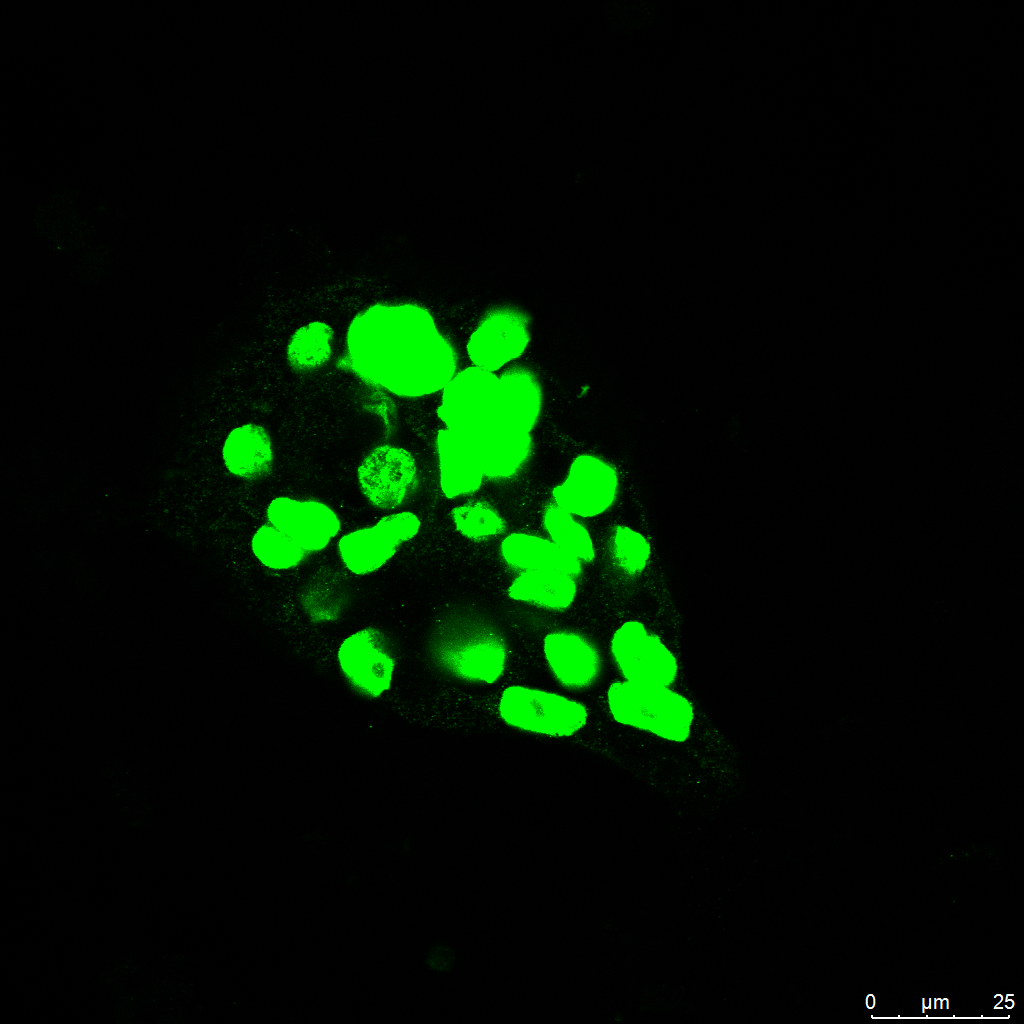

Supplement: Supplementary file 2 — Source data Fig. 1 [file 44319_2025_629_MOESM2_ESM.zip › Figure 1/1H/Left/+DOX 3d GATA3.tif]

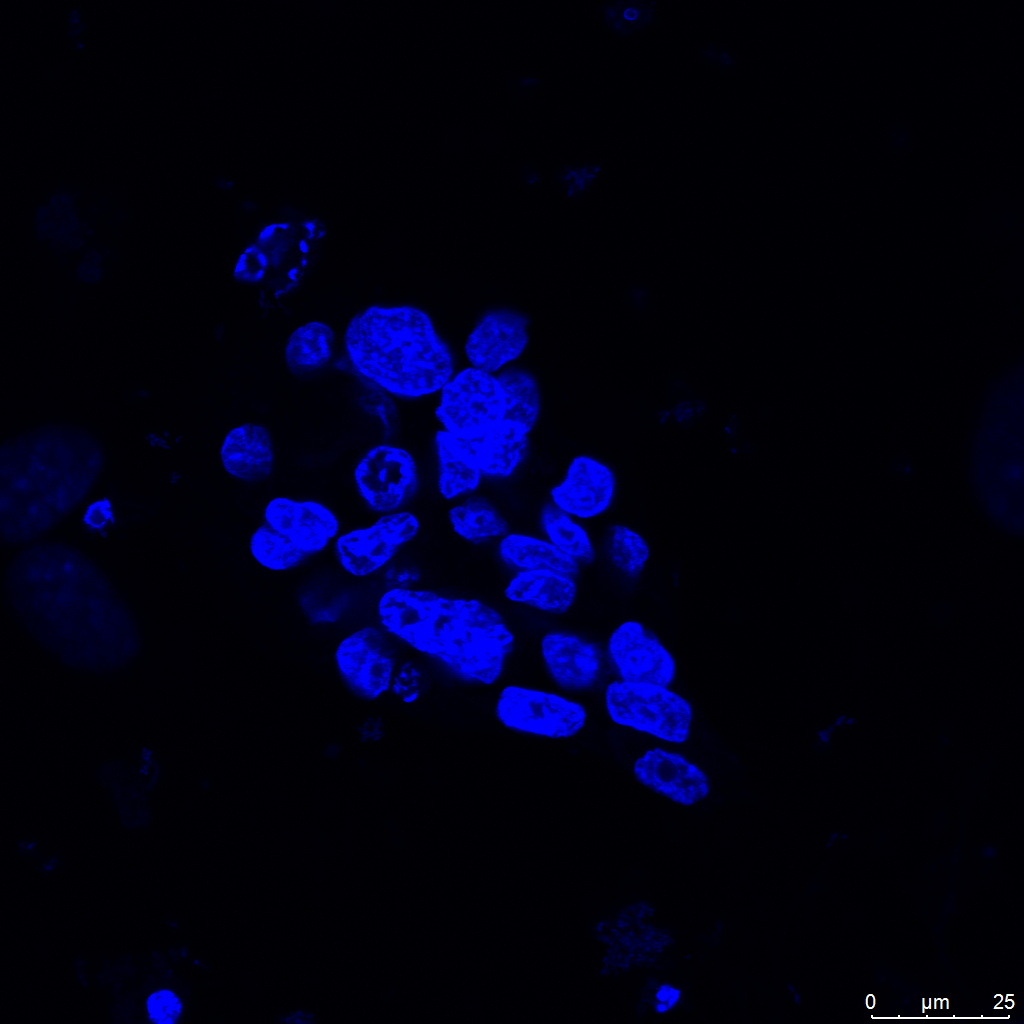

Supplement: Supplementary file 2 — Source data Fig. 1 [file 44319_2025_629_MOESM2_ESM.zip › Figure 1/1H/Left/+DOX 3d Hoechst.tif]

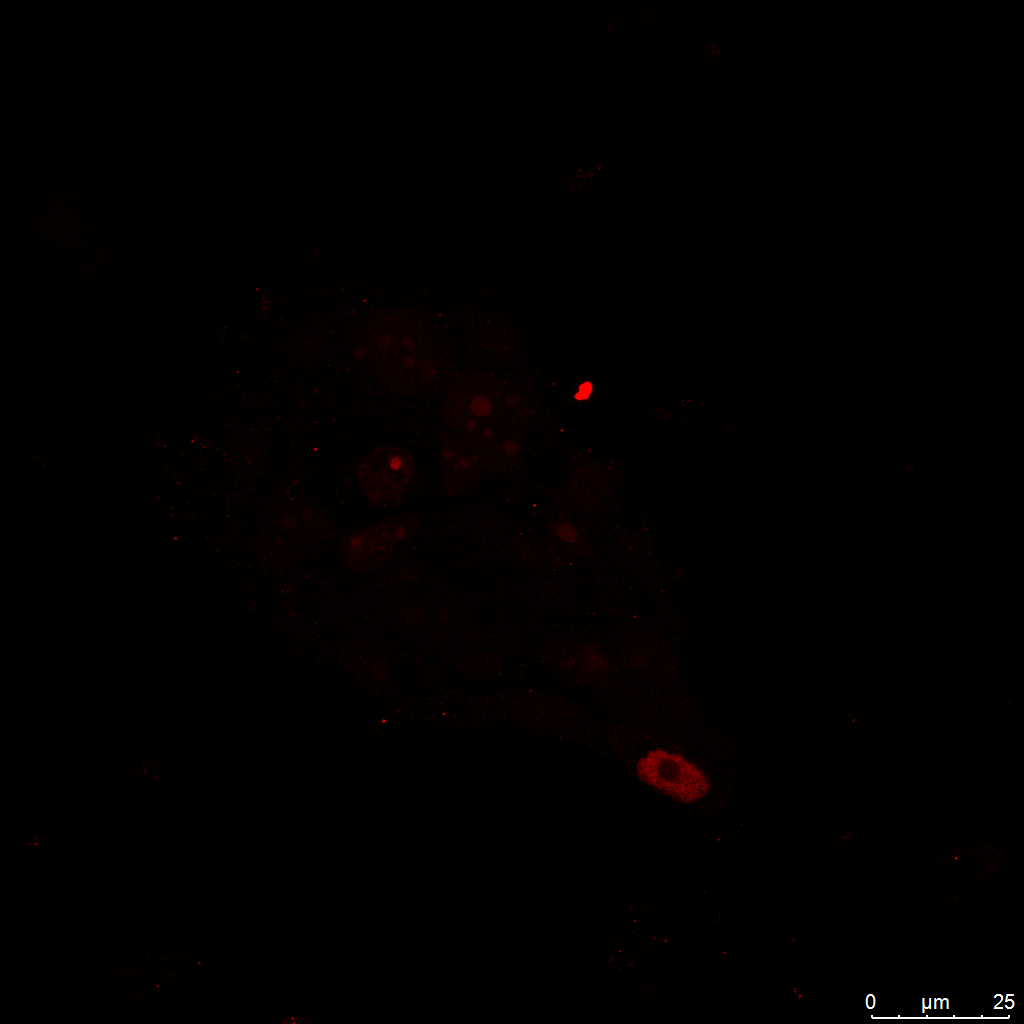

Supplement: Supplementary file 2 — Source data Fig. 1 [file 44319_2025_629_MOESM2_ESM.zip › Figure 1/1H/Left/+DOX 3d NANOG.tif]

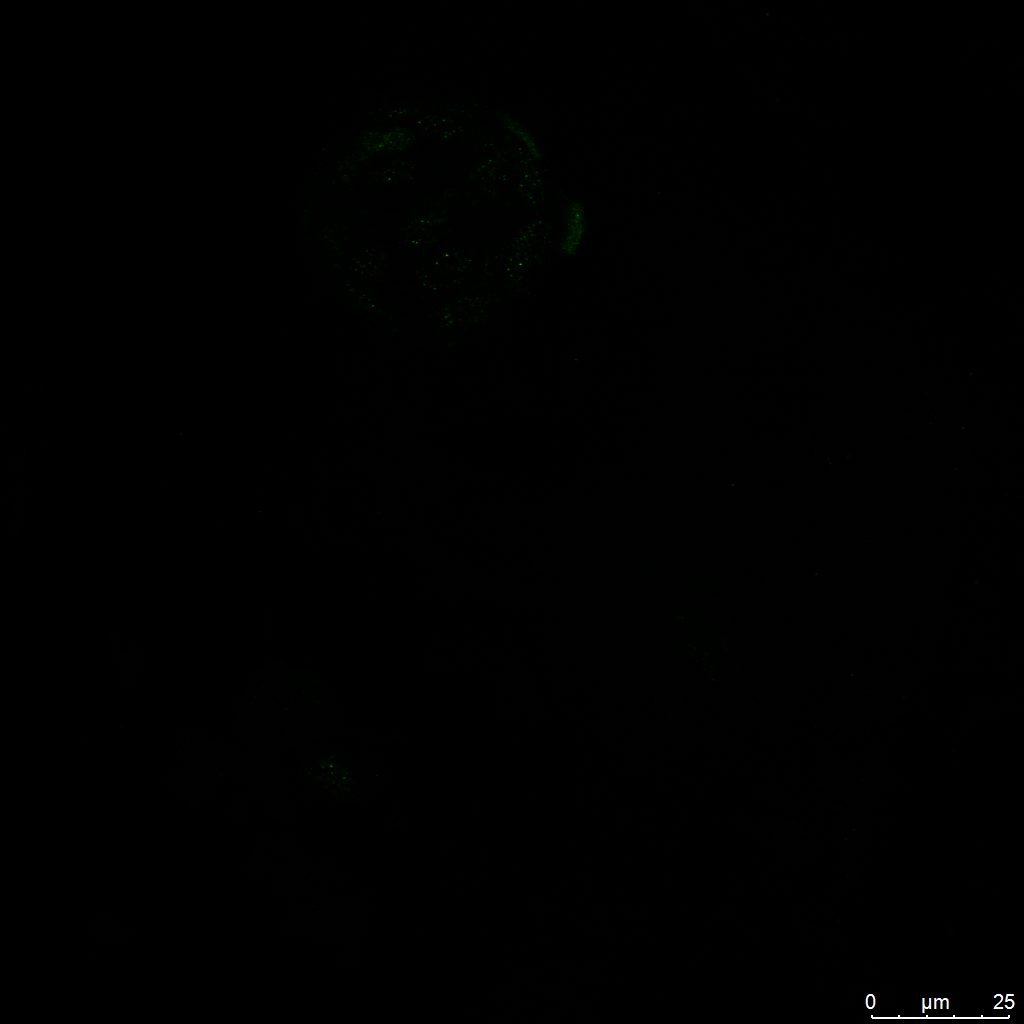

Supplement: Supplementary file 2 — Source data Fig. 1 [file 44319_2025_629_MOESM2_ESM.zip › Figure 1/1H/Left/-DOX GATA3.tif]

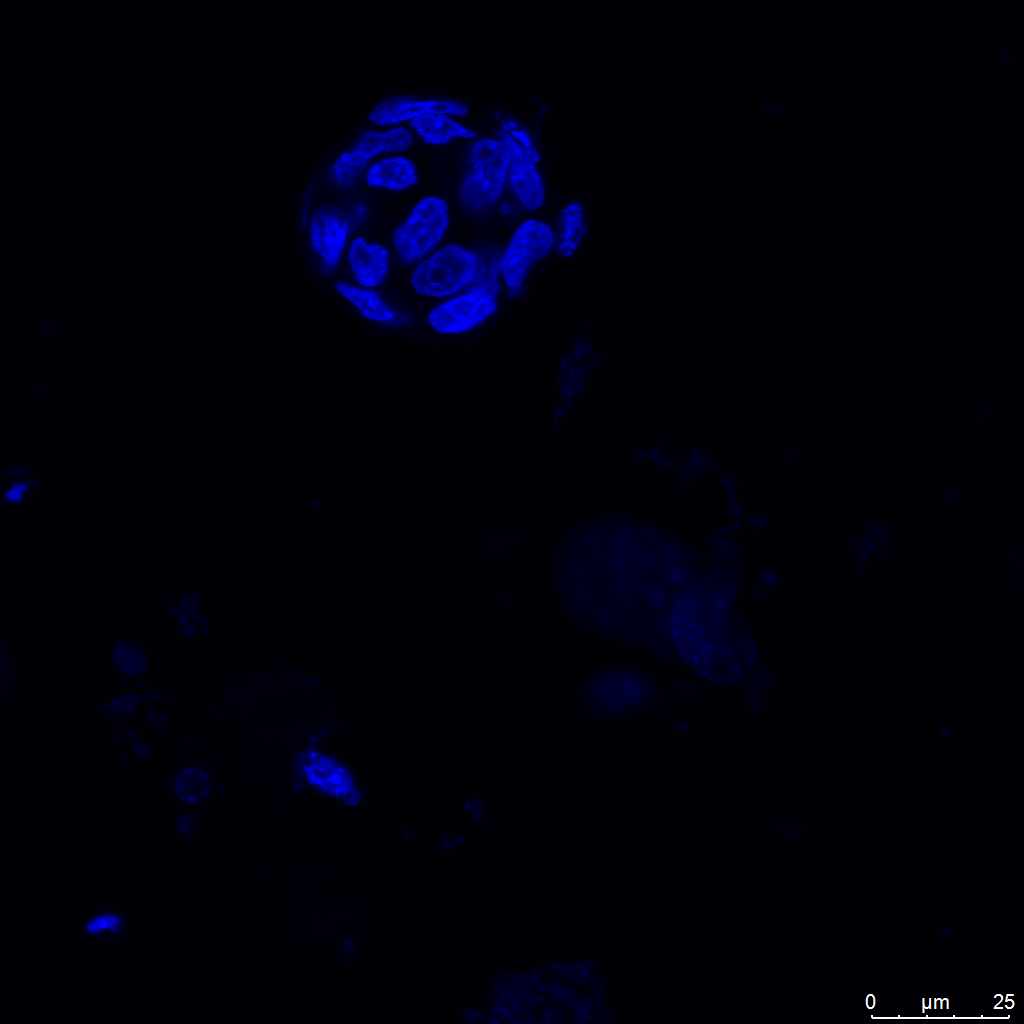

Supplement: Supplementary file 2 — Source data Fig. 1 [file 44319_2025_629_MOESM2_ESM.zip › Figure 1/1H/Left/-DOX Hoechst.tif]

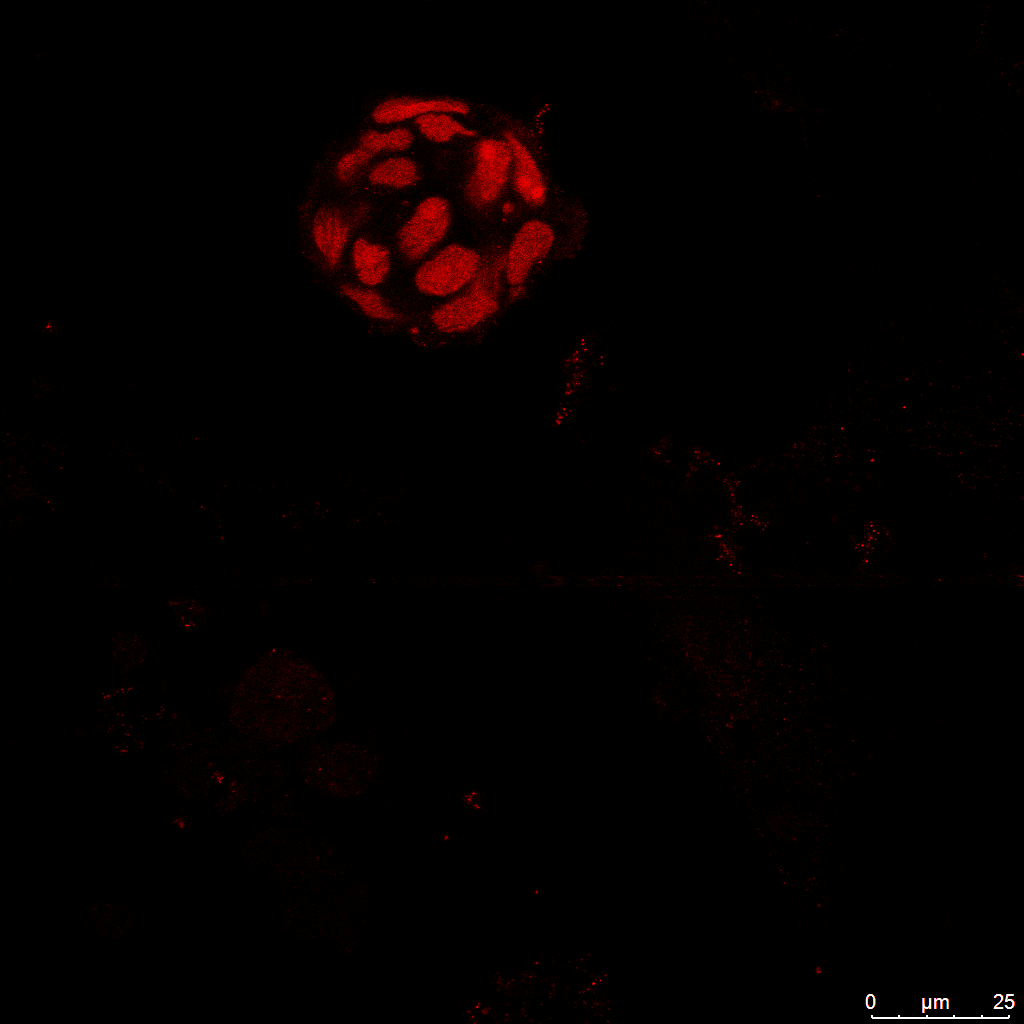

Supplement: Supplementary file 2 — Source data Fig. 1 [file 44319_2025_629_MOESM2_ESM.zip › Figure 1/1H/Left/-DOX NANOG.tif]

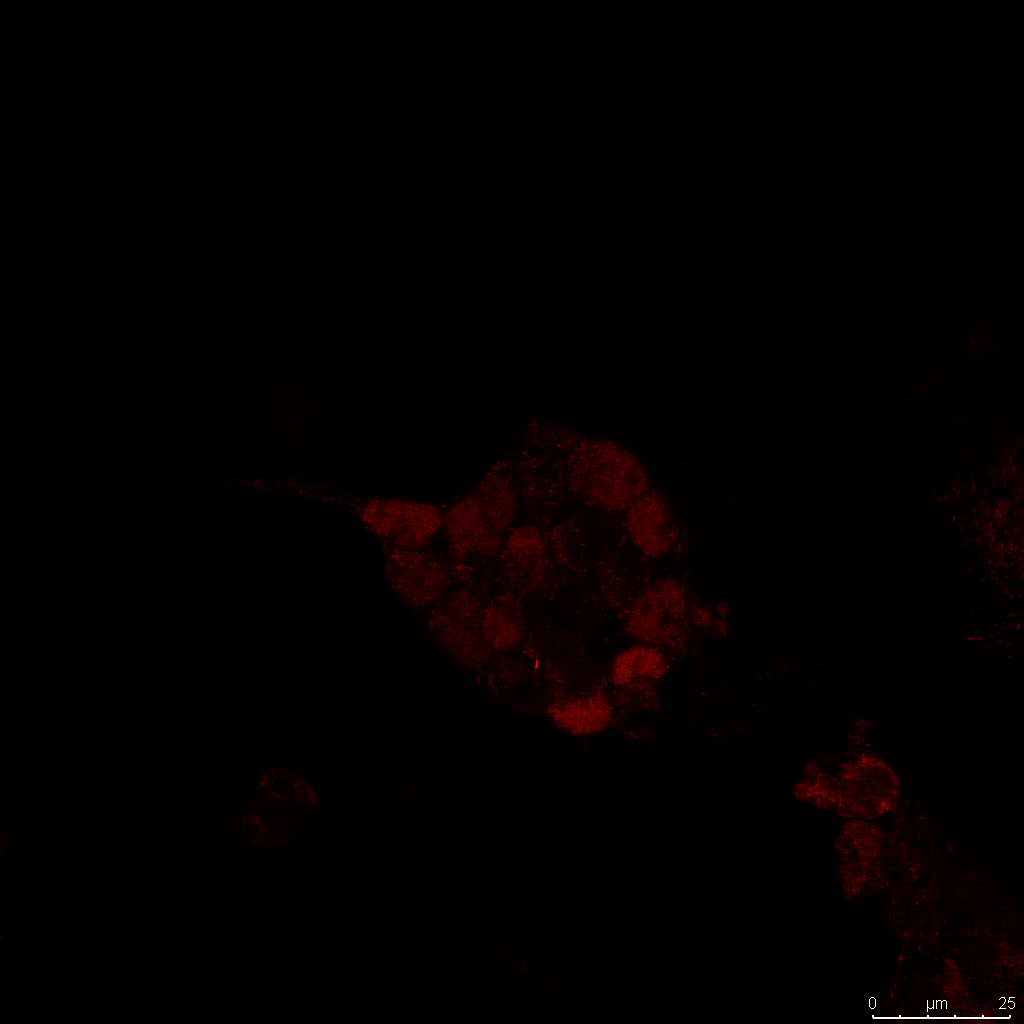

Supplement: Supplementary file 2 — Source data Fig. 1 [file 44319_2025_629_MOESM2_ESM.zip › Figure 1/1H/Right/+DOX 2d GATA6.tif]

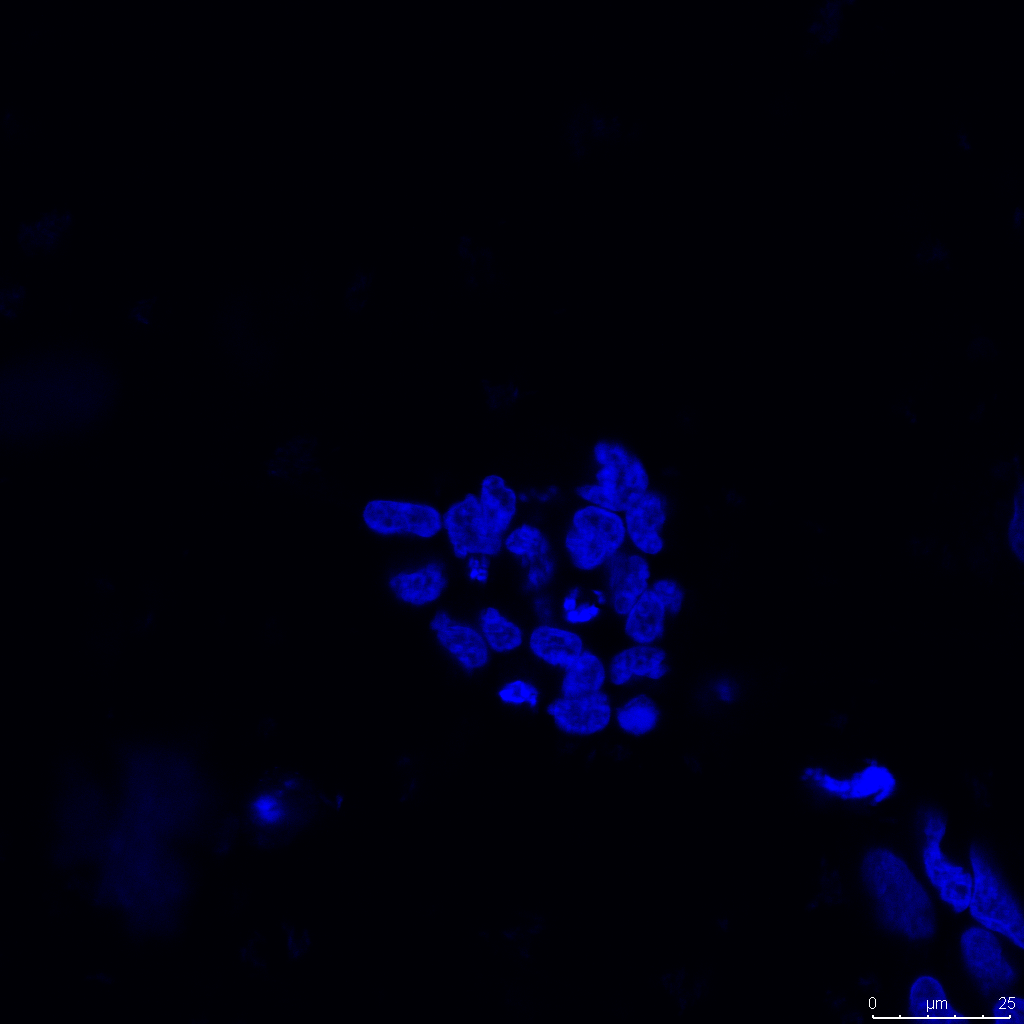

Supplement: Supplementary file 2 — Source data Fig. 1 [file 44319_2025_629_MOESM2_ESM.zip › Figure 1/1H/Right/+DOX 2d Hoechst.tif]

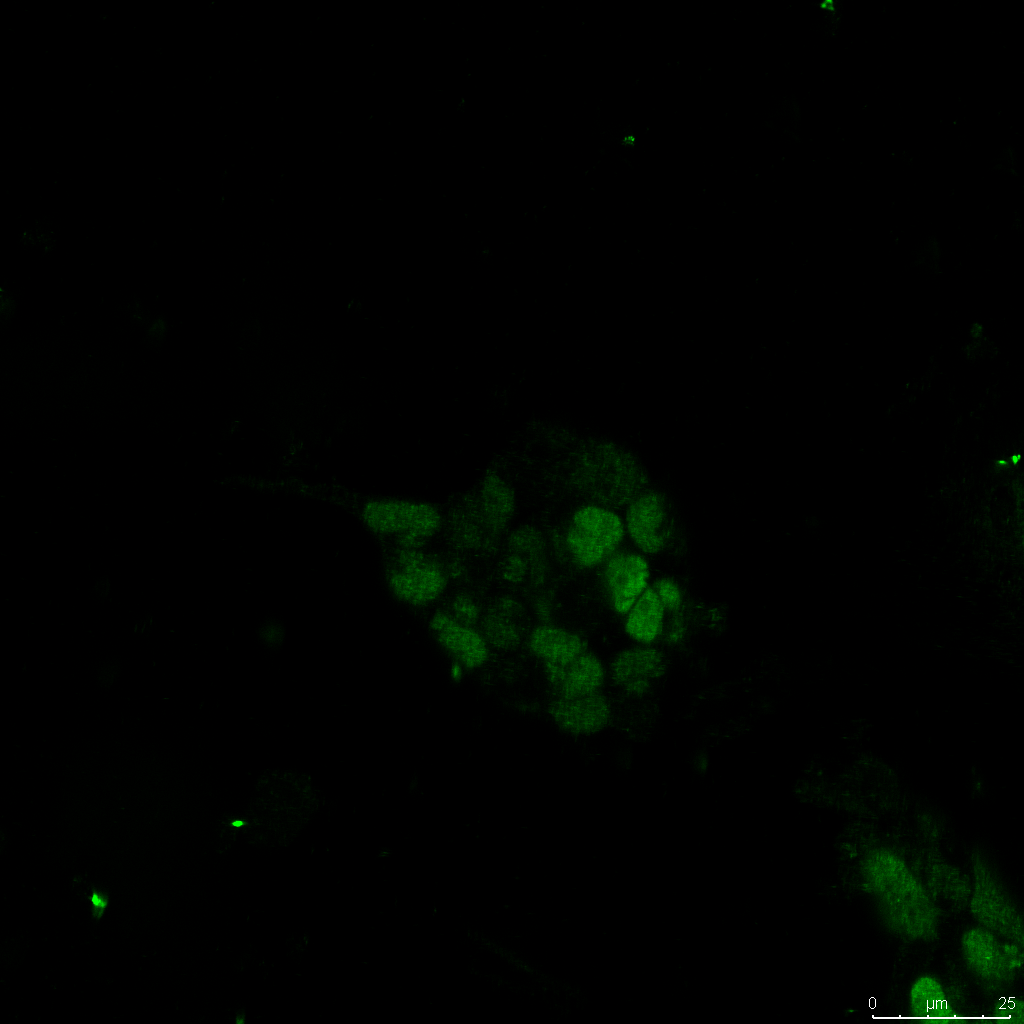

Supplement: Supplementary file 2 — Source data Fig. 1 [file 44319_2025_629_MOESM2_ESM.zip › Figure 1/1H/Right/+DOX 2d NANOG.tif]

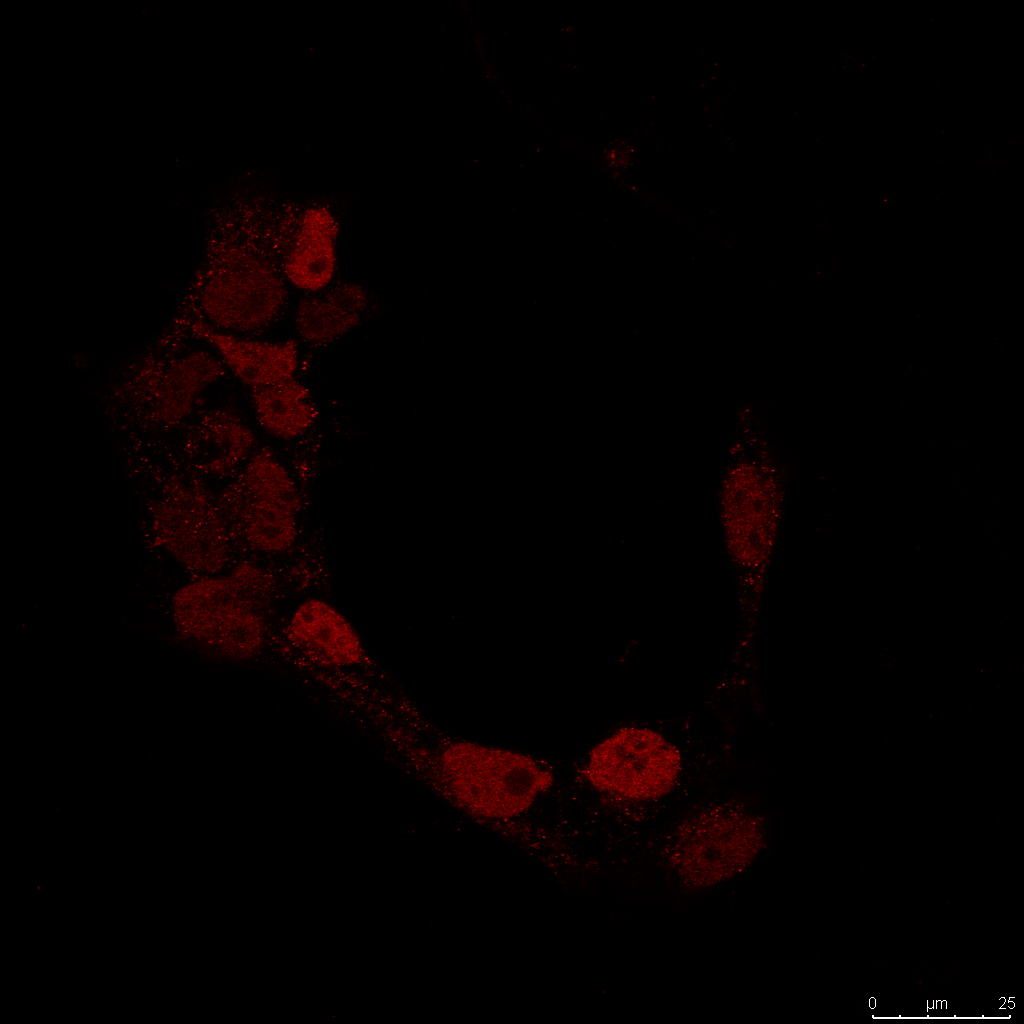

Supplement: Supplementary file 2 — Source data Fig. 1 [file 44319_2025_629_MOESM2_ESM.zip › Figure 1/1H/Right/+DOX 3d GATA6.tif]

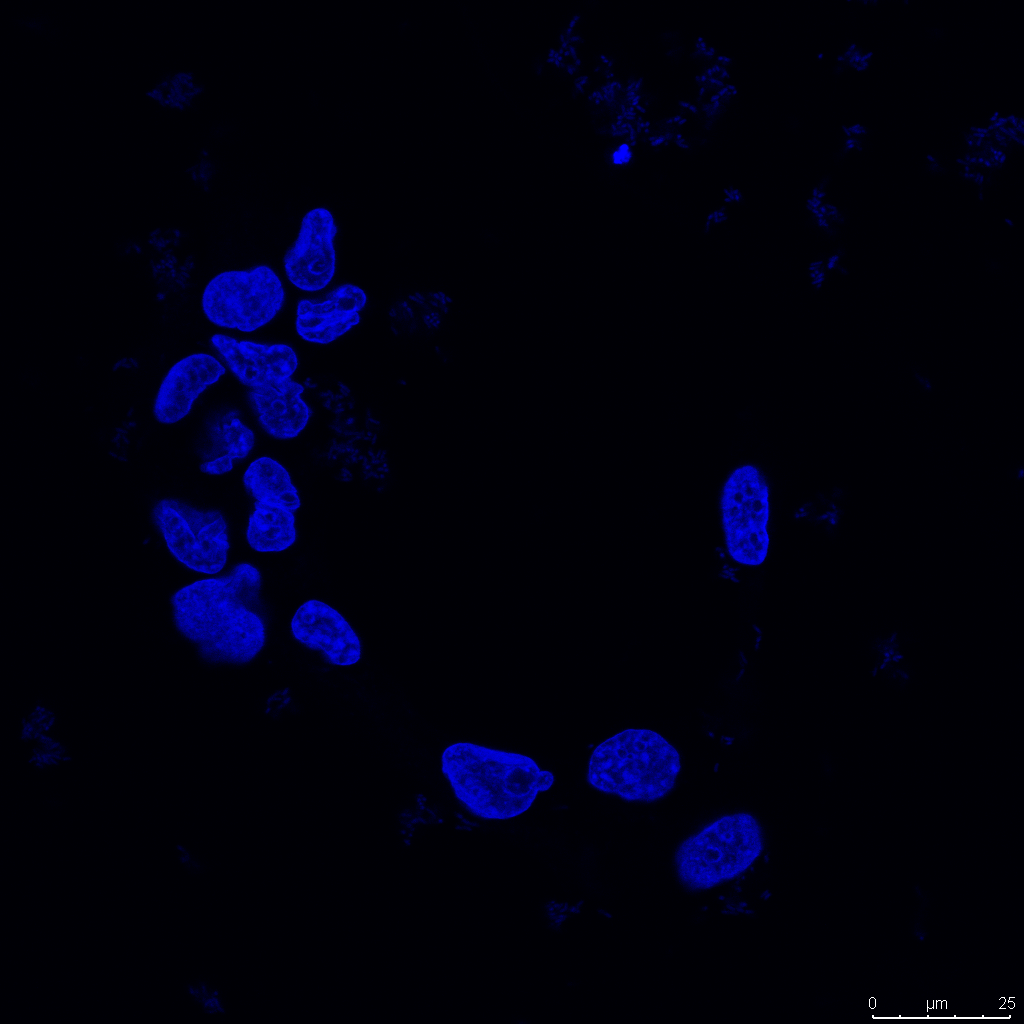

Supplement: Supplementary file 2 — Source data Fig. 1 [file 44319_2025_629_MOESM2_ESM.zip › Figure 1/1H/Right/+DOX 3d Hoechst.tif]

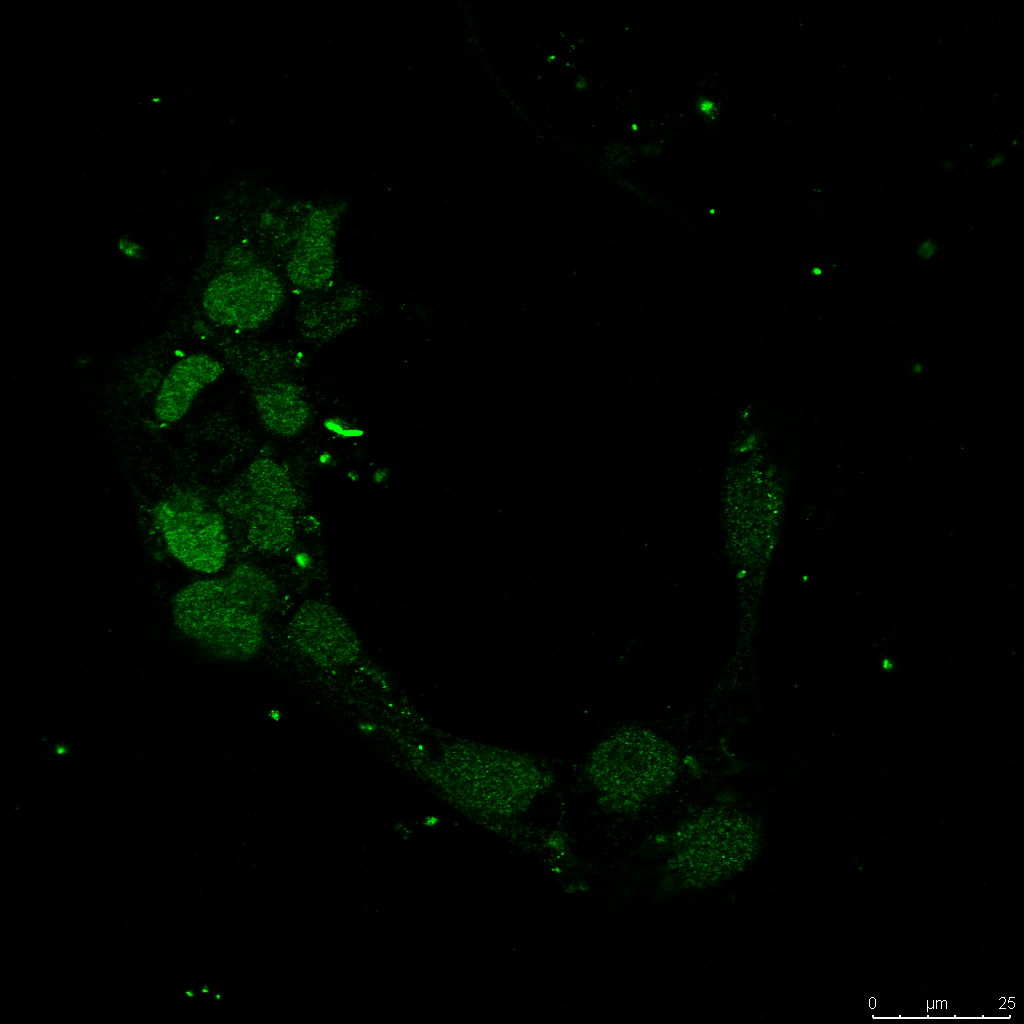

Supplement: Supplementary file 2 — Source data Fig. 1 [file 44319_2025_629_MOESM2_ESM.zip › Figure 1/1H/Right/+DOX 3d NANOG.tif]

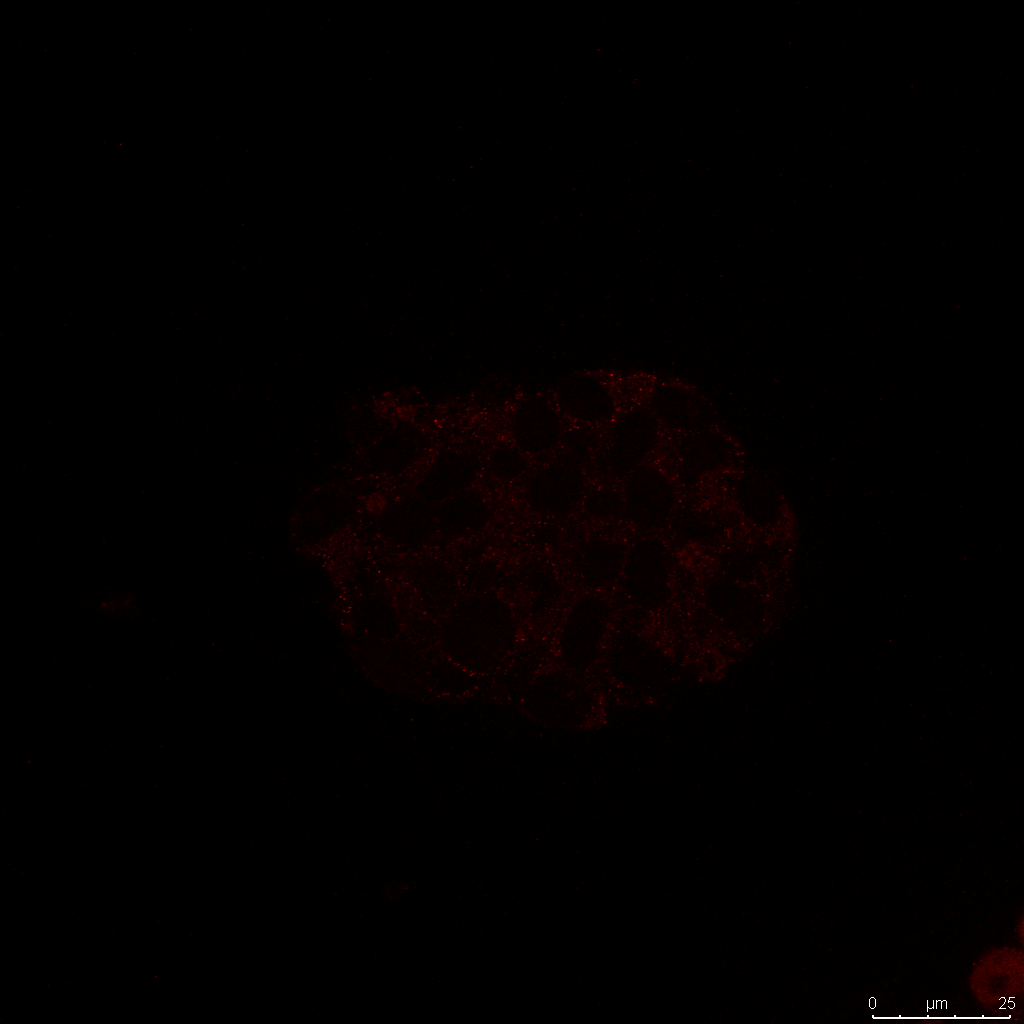

Supplement: Supplementary file 2 — Source data Fig. 1 [file 44319_2025_629_MOESM2_ESM.zip › Figure 1/1H/Right/-DOX GATA6.tif]

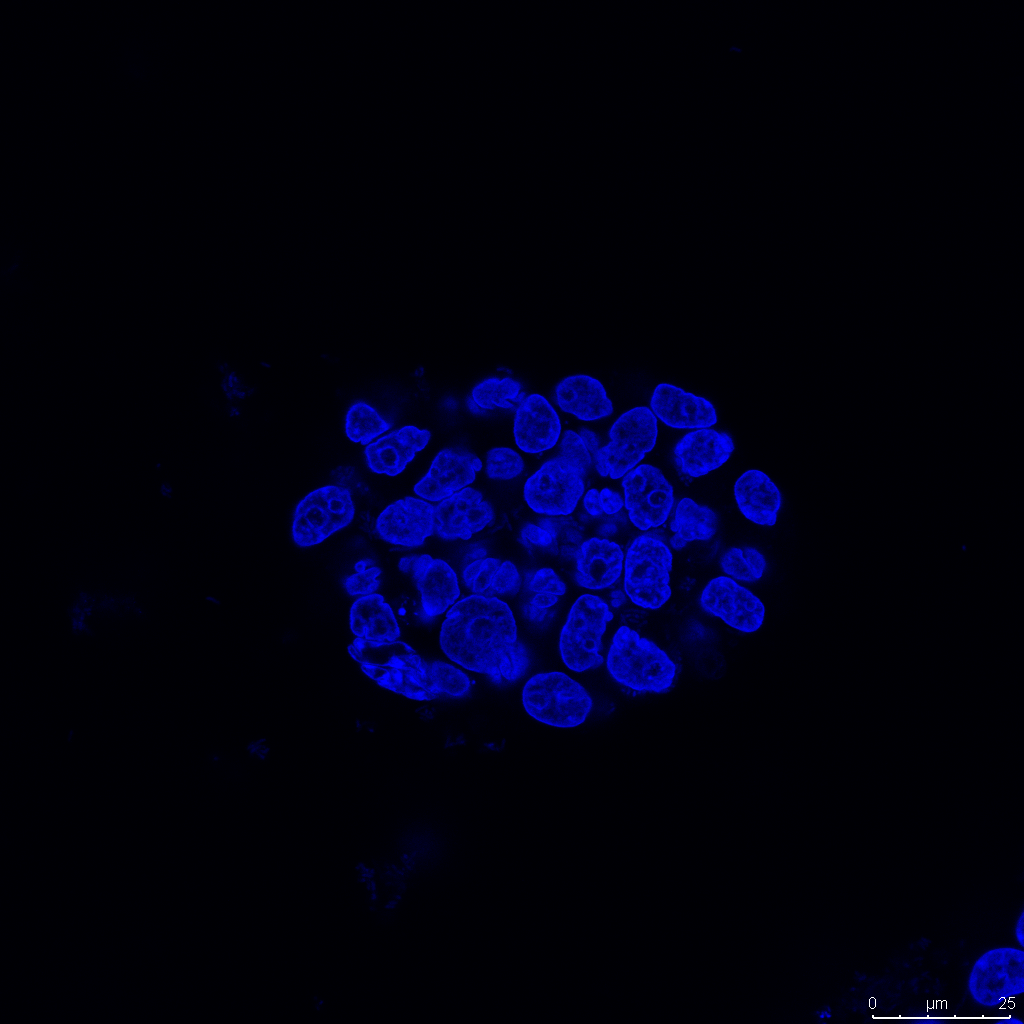

Supplement: Supplementary file 2 — Source data Fig. 1 [file 44319_2025_629_MOESM2_ESM.zip › Figure 1/1H/Right/-DOX Hoechst.tif]

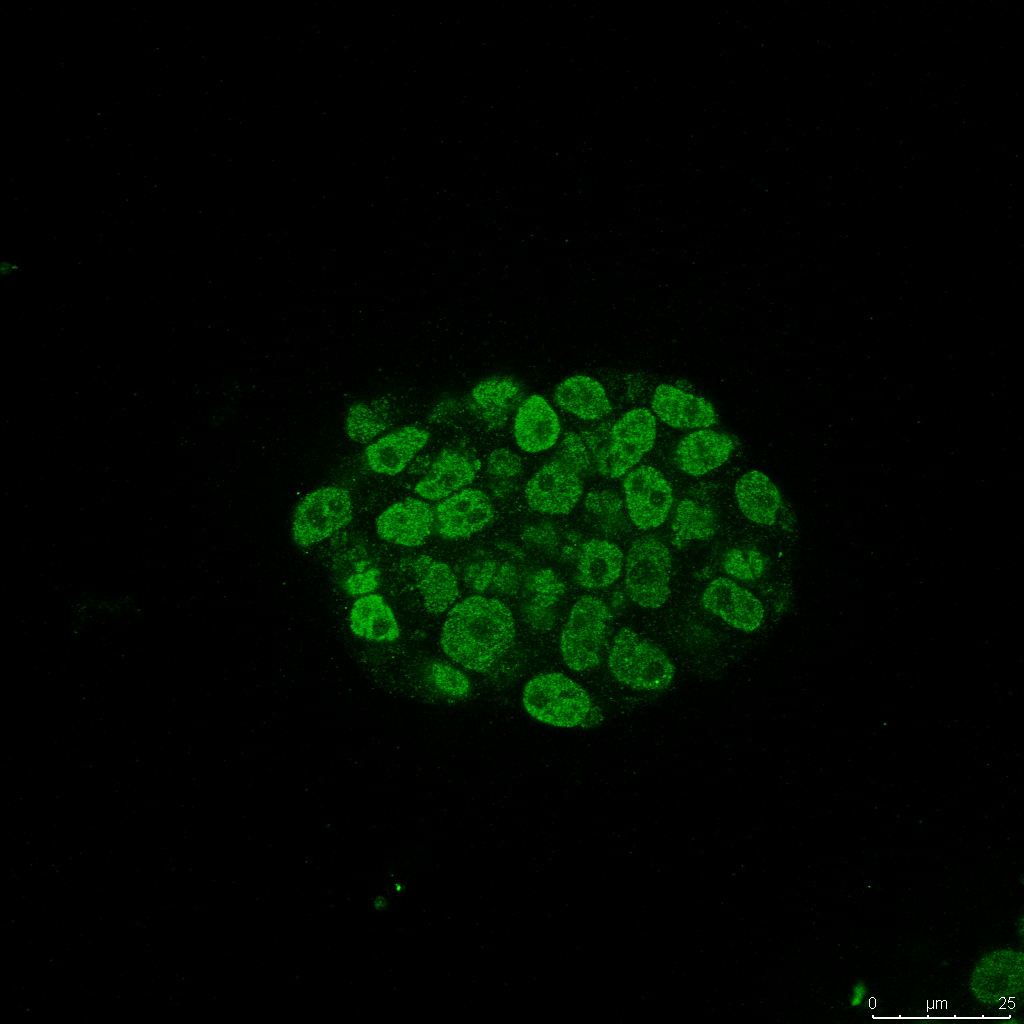

Supplement: Supplementary file 2 — Source data Fig. 1 [file 44319_2025_629_MOESM2_ESM.zip › Figure 1/1H/Right/-DOX NANOG.tif]

Figure 3A

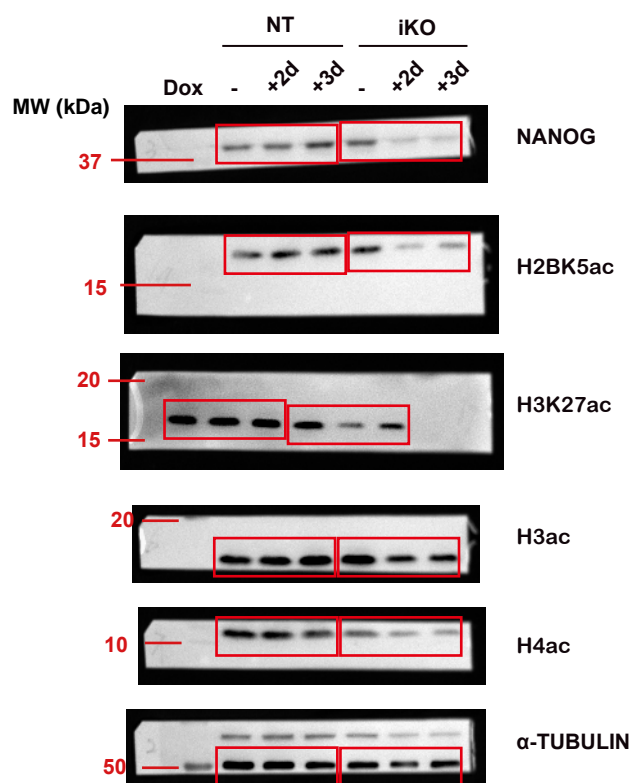

Supplement: Supplementary file 3 — Source data Fig. 3 [file 44319_2025_629_MOESM3_ESM.zip › Figure 3/3A/Fig 3A image.pdf]

Figure 3B

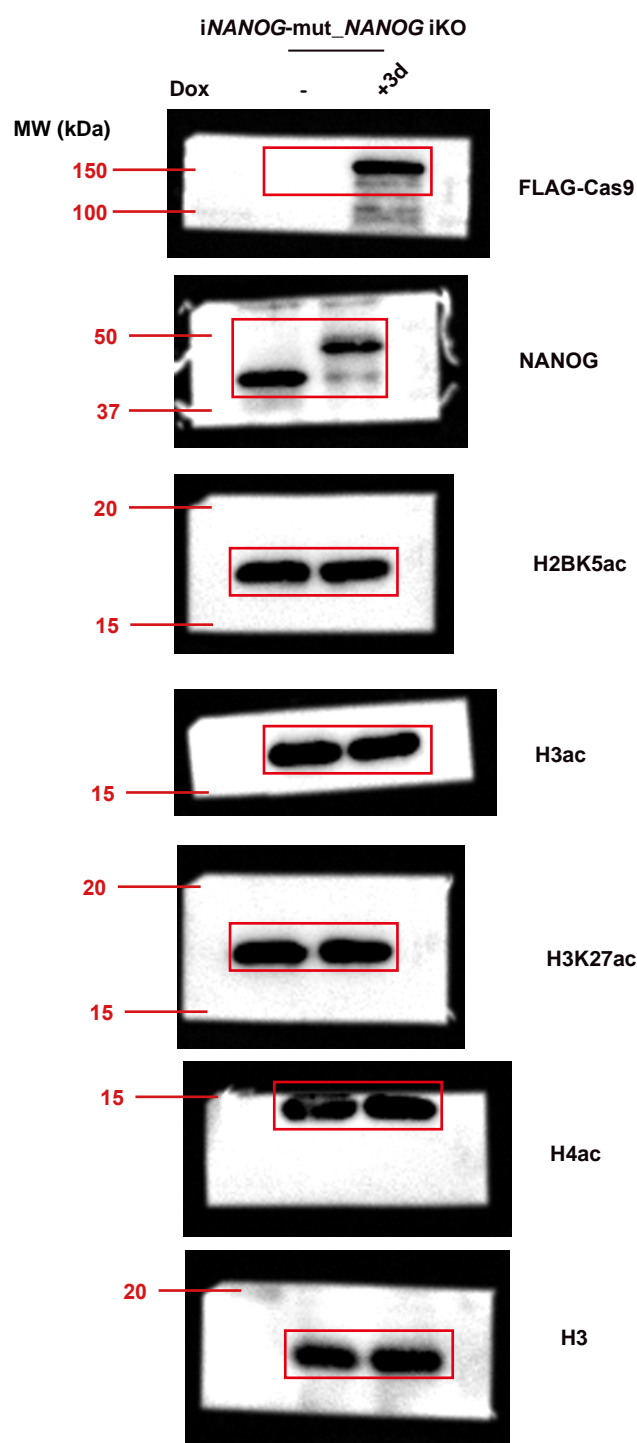

Supplement: Supplementary file 3 — Source data Fig. 3 [file 44319_2025_629_MOESM3_ESM.zip › Figure 3/3B/Fig 3B image.pdf]

Figure 3D

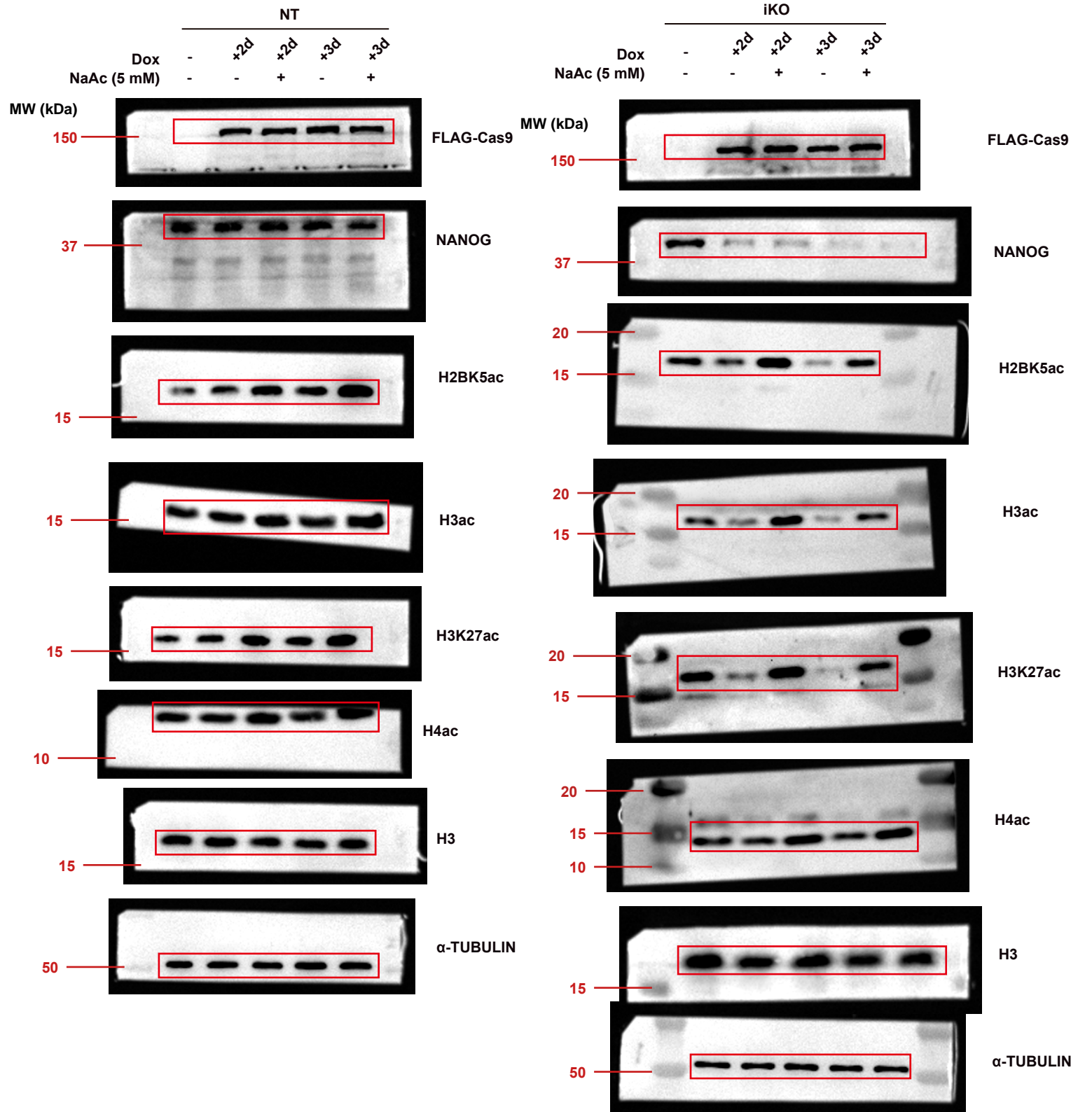

Supplement: Supplementary file 3 — Source data Fig. 3 [file 44319_2025_629_MOESM3_ESM.zip › Figure 3/3D/Fig 3D image.pdf]

Figure 4F

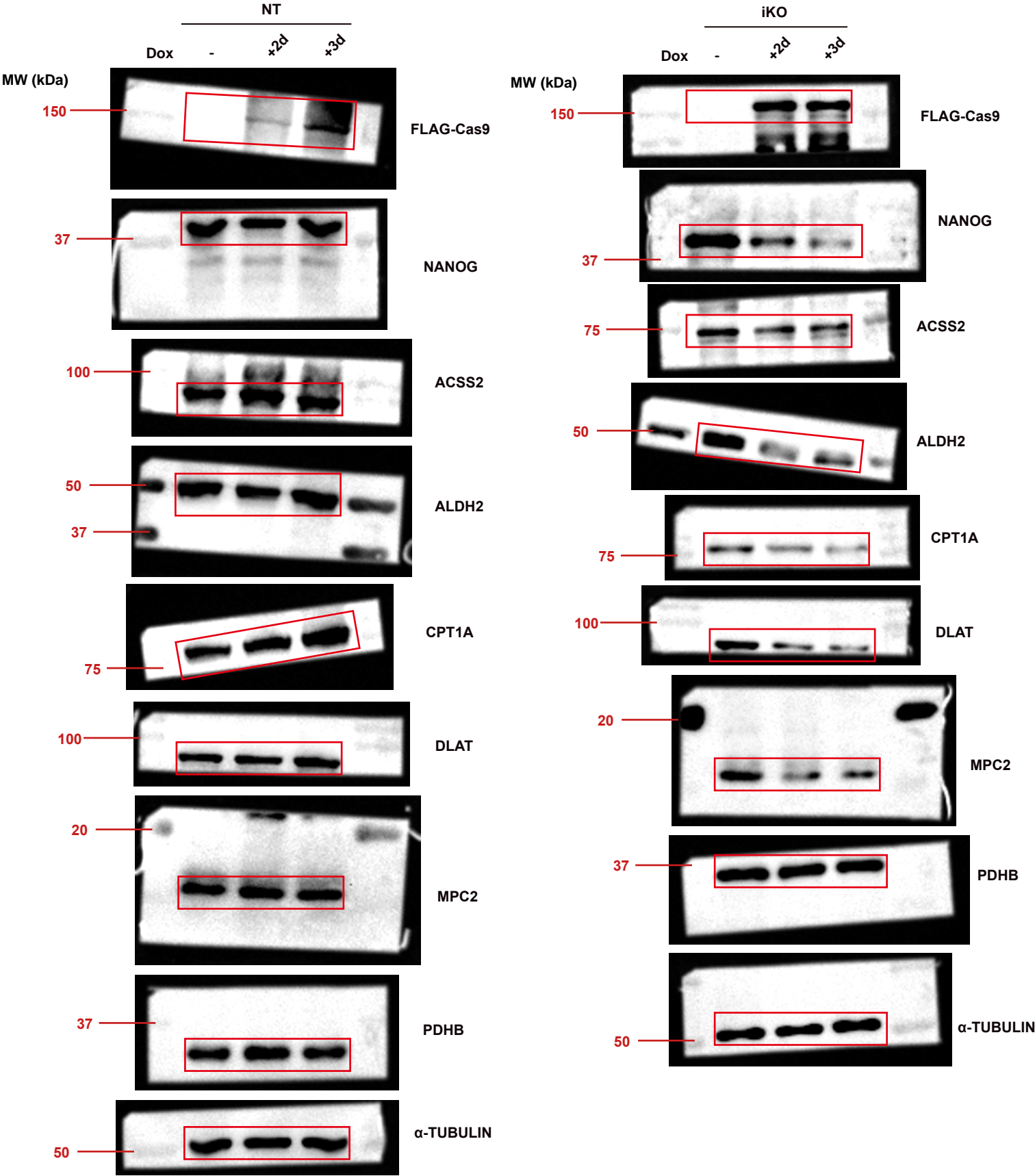

Supplement: Supplementary file 4 — Source data Fig. 4 [file 44319_2025_629_MOESM4_ESM.zip › Figure 4/4F/Fig 4F image.pdf]

Figure 4G

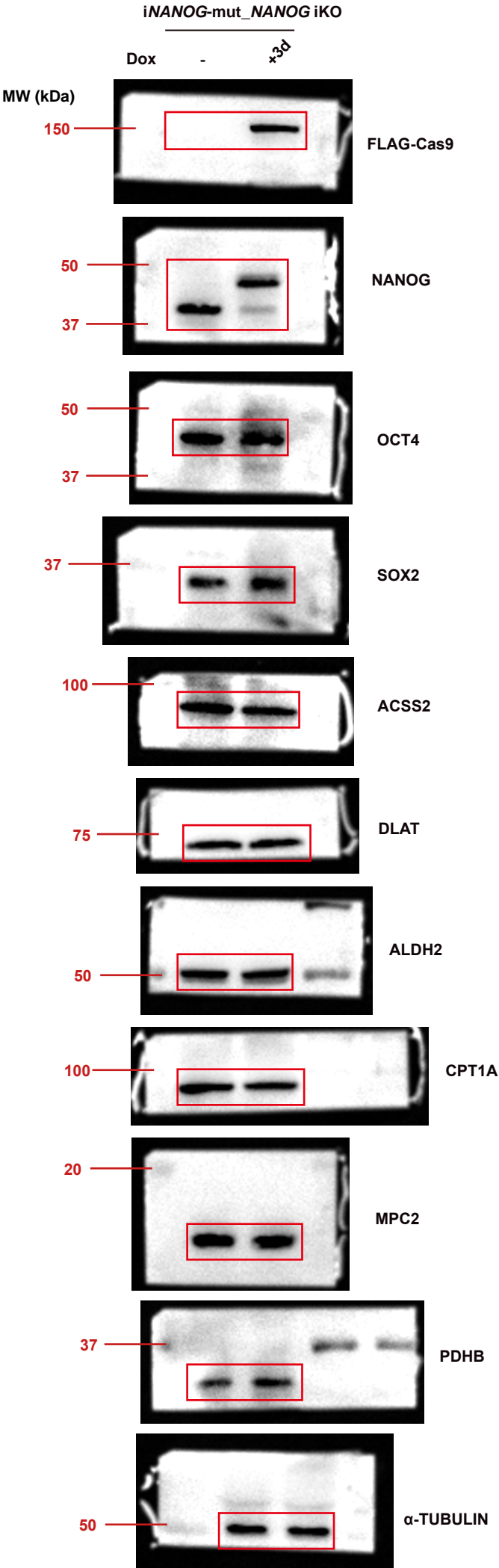

Supplement: Supplementary file 4 — Source data Fig. 4 [file 44319_2025_629_MOESM4_ESM.zip › Figure 4/4G/Fig 4G image.pdf]

Figure 7C

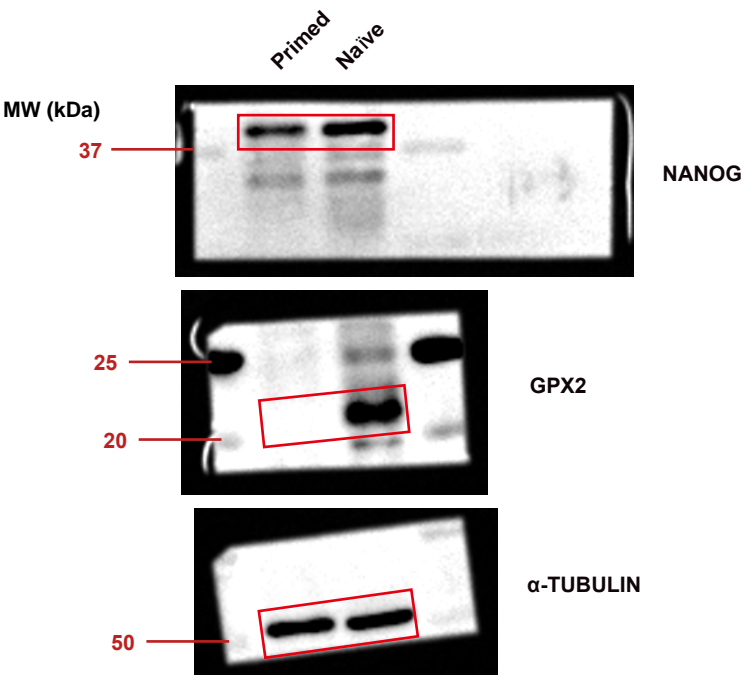

Supplement: Supplementary file 7 — Source data Fig. 7 [file 44319_2025_629_MOESM7_ESM.zip › Figure 7/7C/Fig 7C image.pdf]

### Figure 7D

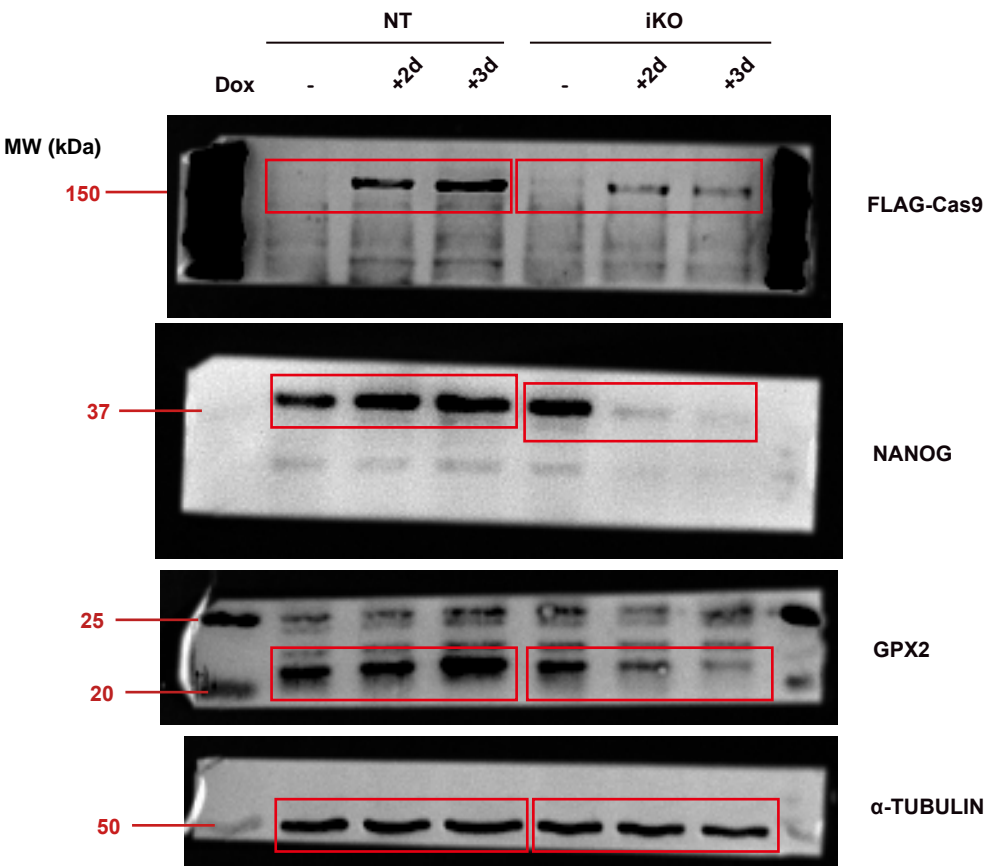

Supplement: Supplementary file 7 — Source data Fig. 7 [file 44319_2025_629_MOESM7_ESM.zip › Figure 7/7D/Fig 7D image.pdf]
