# Supplementary material for: NANOG governs cell metabolism and redox homeostasis in human naïve embryonic stem cells
Source: EMBO Rep. 2025 Nov 19;26(24):6292–324. doi: 10.1038/s44319-025-00629-9 (PMC12714712; doi:10.1038/s44319-025-00629-9)
Supplement: Supplementary file 8 — Expanded View Figures [file 44319_2025_629_MOESM8_ESM.pdf]

Expanded View Figures

Figure EV1. ***NANOG*** **deficiency impairs self-renewal and transcriptional programs of hESCs.**

(**A–C**) Characterization of naïve hESCs by the flow cytometric analysis of the percentage of CD75 and SUSD2 double-positive cells (**A**), RT-qPCR analyses of relative mRNA levels of naïve (top) and primed (bottom) pluripotency markers (**B**), and representative immunofluorescence staining images for NANOG as well as naïve (DNMT3L, KLF17, and REX1) and primed (SSEA4) pluripotency markers in SHhES8 hESCs (**C**). Scale bar, 25 µm. Data are presented as mean ± SEM ($n = 3$) (**A**, **B**). $p = 1.09 \times 10^{-8}$ (**A**). *KLF5*, $p = 1.56 \times 10^{-5}$; *DNMT3L*, $p = 5.76 \times 10^{-5}$; *KHDC3L*, $p = 1.33 \times 10^{-5}$ (**B**). (**D**) The representative western blot analysis result showing protein levels of NANOG and cleaved Caspase-3 in naïve (top) and primed (bottom) NT or *NANOG* iKO SHhES8 hESCs, either untreated or treated with DOX for the indicated time lengths. Alpha-TUBULIN served as a loading control. (**E**) Representative phase contrast images displaying cell colonies of primed NT (left) and *NANOG* iKO (right) SHhES8 hESCs, either untreated or treated with DOX for two or three days. (**F**) The line chart depicting live cell counts of primed NT (top) or *NANOG* iKO (bottom) SHhES8 hESCs, either untreated or treated with DOX over the indicated days. Data are presented as mean ± SEM ($n = 3$). (**G**, **H**) Volcano plots depicting upregulated or downregulated DEGs in naïve (**G**) and primed (**H**) *NANOG* iKO SHhES8 hESCs induced by *NANOG* depletion. Upregulated genes (red dots) were defined by an adjusted *p* value (*p*adj) <0.05 and log2 (FoldChange) >0; Downregulated genes (blue dots) are shown with *p*adj <0.05 and log2 (FoldChange) <0. Comparisons were made between DOX-treated cells and their untreated counterparts. NS, not significantly changed. DESeq2 was used for the statistical analysis of DEGs. Three biological replicates were used for each condition ($n = 3$). (**I**) Heatmaps displaying normalized RNA-seq levels of selected markers for three germ layers and primed pluripotency state in primed *NANOG* iKO SHhES8 hESCs, either untreated or treated with DOX for two or three days. The color represents Z-scores. Three biological replicates were used for each condition ($n = 3$). The unpaired two-tailed Student's *t*-test was used for the statistical analysis in (**A**, **B**, **F**).

© The Author(s)

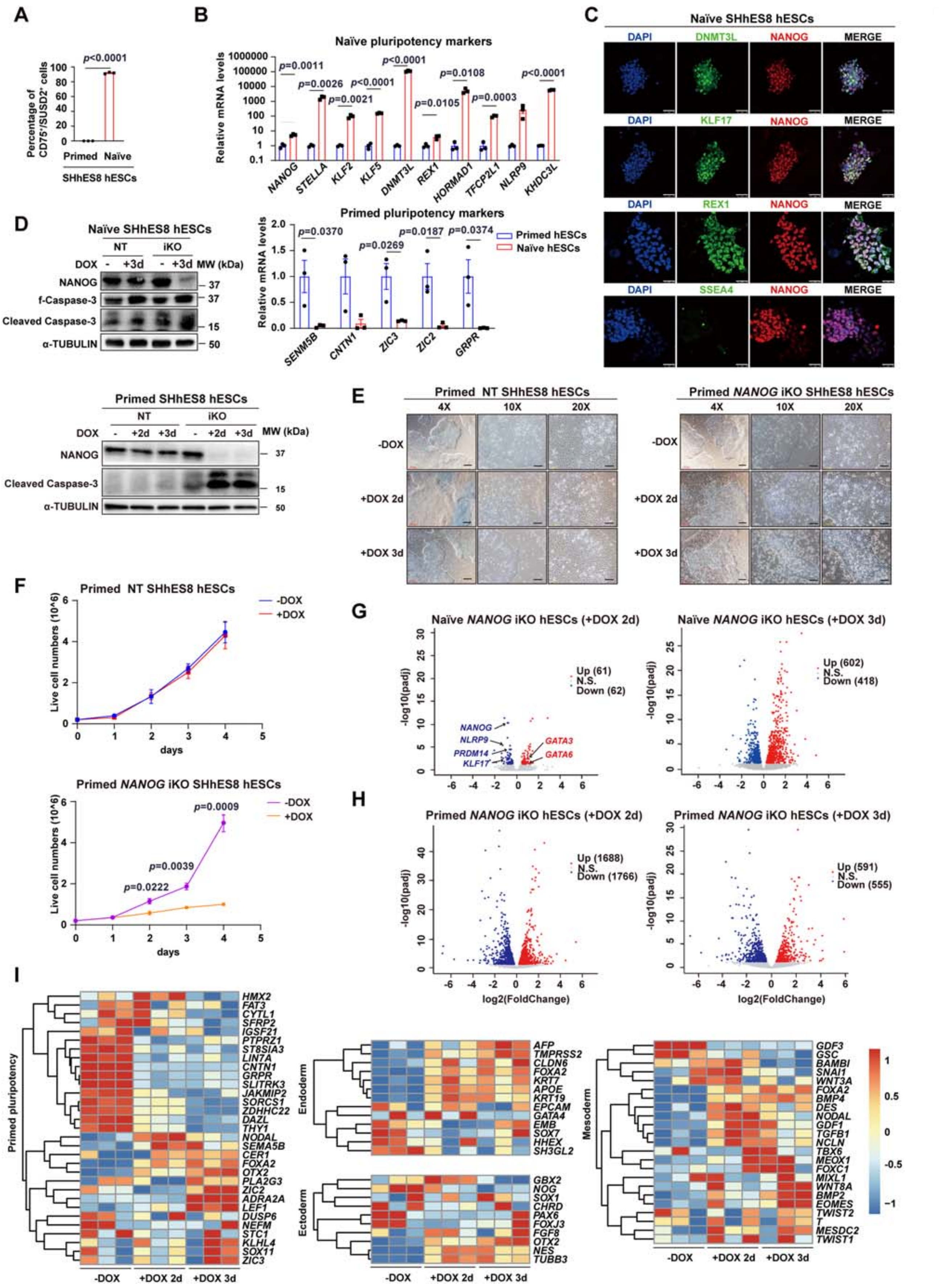
A
p<0.0001
Percentage of CD75+/SUSD2+ cells
Primed
Naïve
SHhES8 hESCs
B
Naïve pluripotency markers
Relative mRNA levels
p=0.0011
p=0.0026
p=0.0021
p<0.0001
p<0.0001
p=0.0105
p=0.0108
p=0.0003
p<0.0001
NANOG
STELLA
KLF2
KLF5
DNMT3L
REX1
HORMAD1
TFCP2L1
NLRP9
KHDC3L
Primed pluripotency markers
p=0.0370
p=0.0269
p=0.0187
p=0.0374
Primed hESCs
Naïve hESCs
SENM5B
CNTN1
ZIC3
ZIC2
GRPR
C
Naïve SHhES8 hESCs
DAPI
DNMT3L
NANOG
MERGE
KLF17
REX1
SSEA4
D
Naïve SHhES8 hESCs
NT
iKO
DOX
+3d
MW (kDa)
NANOG
f-Caspase-3
Cleaved Caspase-3
α-TUBULIN
Primed SHhES8 hESCs
+2d
E
Primed NT SHhES8 hESCs
Primed NANOG iKO SHhES8 hESCs
4X
10X
20X
-DOX
+DOX 2d
+DOX 3d
F
Primed NT SHhES8 hESCs
Live cell numbers (10^6)
days
-DOX
+DOX
Primed NANOG iKO SHhES8 hESCs
p=0.0009
p=0.0039
p=0.0222
G
Naïve NANOG iKO hESCs (+DOX 2d)
Naïve NANOG iKO hESCs (+DOX 3d)
-log10(padj)
Up (61)
N.S.
Down (62)
NANOG
NLRP9
PRDM14
KLF17
GATA3
GATA6
Up (602)
Down (418)
H
Primed NANOG iKO hESCs (+DOX 2d)
Primed NANOG iKO hESCs (+DOX 3d)
Up (1688)
Down (1766)
Up (591)
Down (555)
log2(FoldChange)
I
Primed pluripotency
HMX2
FAT3
CYTL1
SFRP2
IGSF21
PTPRZ1
ST8SIA3
LIN7A
CNTN1
GRPR
SLITRK3
JAKMIP2
SORCS1
ZDHHC22
DAZL
THY1
NODAL
SEMA5B
CER1
FOXA2
OTX2
PLA2G3
ZIC2
ADRA2A
LEF1
DUSP6
NEFM
STC1
KLHL4
SOX11
ZIC3
Endoderm
AFP
TMPRSS2
CLDN6
FOXA2
KRT7
APOE
KRT19
EPCAM
GATA4
EMB
SOX7
HHEX
SH3GL2
Ectoderm
GBX2
NOG
SOX1
CHRD
PAX6
FOXJ3
FGF8
OTX2
NES
TUBB3
Mesoderm
GDF3
GSC
BAMBI
SNAI1
WNT3A
FOXA2
BMP4
DES
NODAL
GDF1
TGFB1
NCLN
TBX6
MEOX1
FOXC1
MIXL1
WNT8A
BMP2
EOMES
TWIST2
T
MESDC2
TWIST1
-DOX
+DOX 2d
+DOX 3d

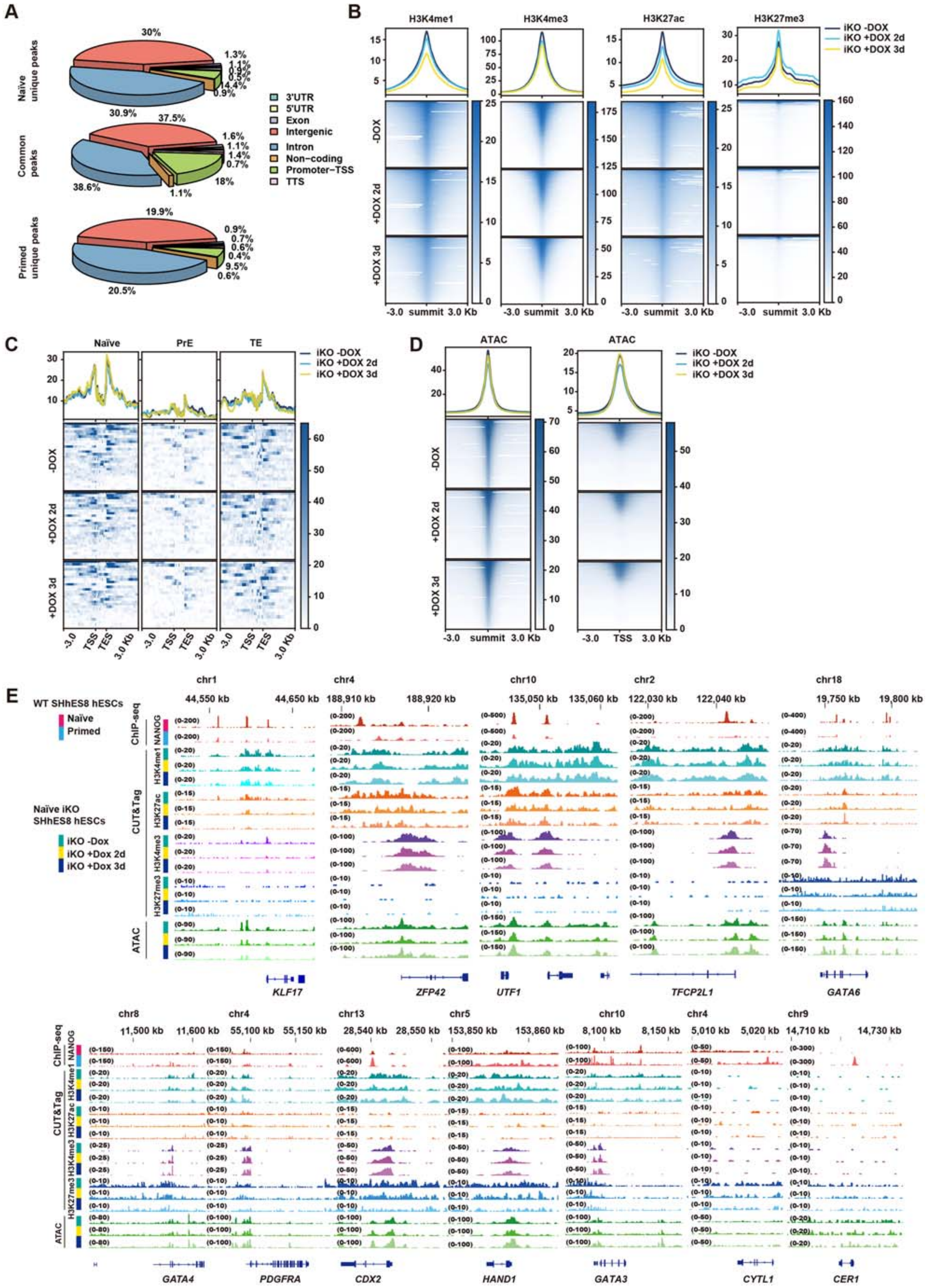

 © The Author(s)

Figure EV2. ***NANOG*** **depletion results in distinct epigenetic changes for marker genes of different lineages.**

(**A**) Pie charts displaying the genomic distribution of the three groups of NANOG binding peaks. (**B**) Heatmaps depicting changes in the abundance (normalized by total reads) of histone modifications (H3K27ac, H3K27me3, H3K4me1, and H3K4me3) across the whole genome in naïve *NANOG* iKO SHhES8 hESCs, either untreated or treated with DOX for two or three days, as analyzed by the deepTools. (**C**, **D**) Heatmaps illustrating the abundance (normalized by total reads) of ATAC-seq signals within a 3 kb range upstream and downstream of marker genes associated with naïve pluripotency, TE, or PrE (**C**), and of ATAC-seq signals across the whole genome (left) or TSS regions (right) (**D**) in naïve *NANOG* iKO SHhES8 hESCs, either untreated or treated with DOX for three days, as analyzed by the deepTools. (**E**) Genome browser snapshots of NANOG occupancy at the vicinity of naïve pluripotency markers (*KLF17, ZFP42, UTF1*, and *TFCP2L1*), PrE markers (*GATA6, GATA4*, and *PDGFRA*), TE markers (*GATA3, CDX2*, and *HAND1*), and primed pluripotency markers (*CYTL1* and *CER1*) in primed and naïve SHhES8 hESCs, as well as histone modifications and chromatin openness in naïve *NANOG* iKO SHhES8 hESCs, either untreated or treated with DOX for two or three days.

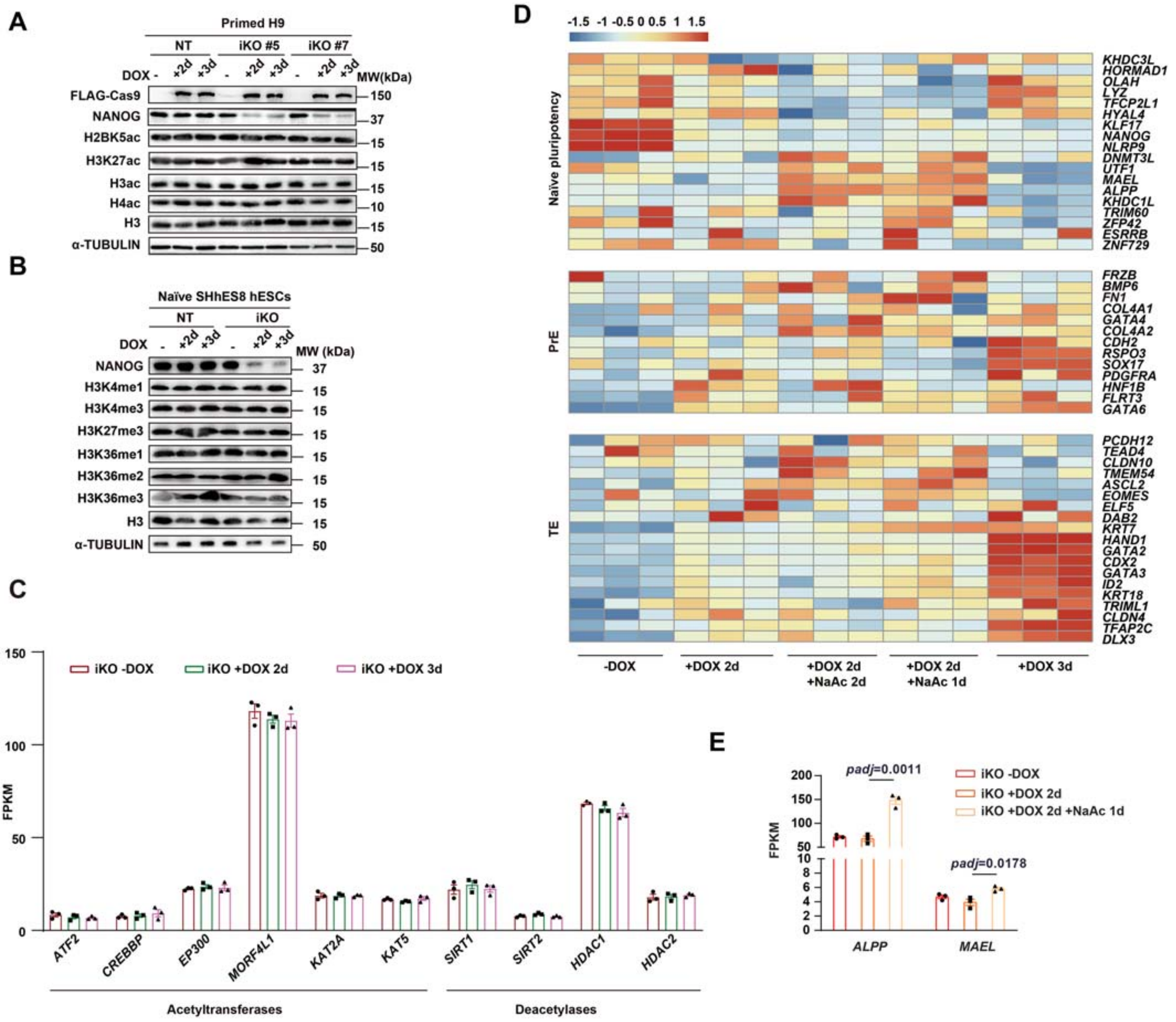

Figure EV3. NANOG maintains histone acetylation modifications via controlling acetyl-CoA production in naïve hESCs.

(**A**) The representative western blot analysis result for levels of NANOG proteins and histone acetylation modifications in primed NT and *NANOG* iKO H9 hESCs, either untreated or treated with DOX for two or three days. Alpha-TUBULIN and histone 3 served as loading controls. Single clonal culture of *NANOG* iKO #5 and #7 was used. (**B**) The representative western blot analysis result for levels of NANOG and histone methylation modifications in naïve NT and *NANOG* iKO SHhES8 hESCs, either untreated or treated with DOX for two or three days. Alpha-TUBULIN and histone 3 served as loading controls. (**C**) The FPKM values from our RNA sequencing data for a set of genes related to histone acetylation modifications in naïve *NANOG* iKO hESCs, either untreated or treated with DOX for two or three days. Data are presented as mean ± SEM ($n = 3$). The unpaired two-tailed student's *t*-test was used for statistical analysis. (**D**) Heatmaps showing normalized mRNA levels, measured by our RNA-seq assay, of selected naïve pluripotency, PrE and TE marker genes in naïve *NANOG* iKO SHhES8 hESCs cultured under indicated conditions. The color represents Z-scores. Three biological replicates were used for each culture condition. (**E**) The FPKM values from our RNA sequencing data for two naïve pluripotency marker genes, *ALPP* and *MAEL*, either untreated or treated with DOX or DOX and NaAc in naïve *NANOG* iKO hESCs. Data are presented as mean ± SEM ($n = 3$). DESeq2 was used for the statistical analysis.

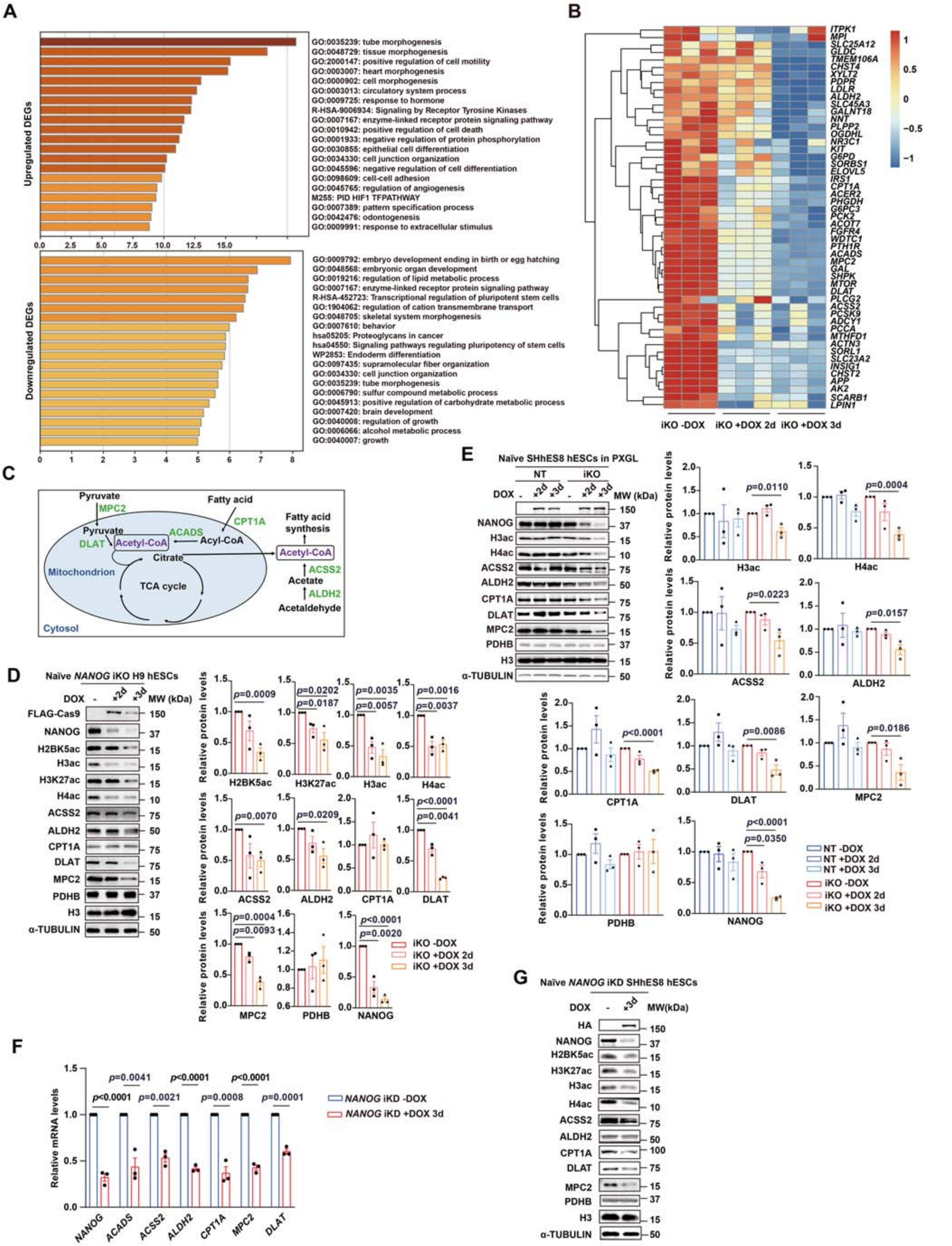

A
Upregulated DEGs
GO:0035239: tube morphogenesis
GO:0048729: tissue morphogenesis
GO:2000147: positive regulation of cell motility
GO:0003007: heart morphogenesis
GO:0000902: cell morphogenesis
GO:0003013: circulatory system process
GO:0009725: response to hormone
R-HSA-9006934: Signaling by Receptor Tyrosine Kinases
GO:0007167: enzyme-linked receptor protein signaling pathway
GO:0010942: positive regulation of cell death
GO:0001933: negative regulation of protein phosphorylation
GO:0030855: epithelial cell differentiation
GO:0034330: cell junction organization
GO:0045596: negative regulation of cell differentiation
GO:0098609: cell-cell adhesion
GO:0045765: regulation of angiogenesis
M255: PID HIF1 TFPATHWAY
GO:0007389: pattern specification process
GO:0042476: odontogenesis
GO:0009991: response to extracellular stimulus
Downregulated DEGs
GO:0009792: embryo development ending in birth or egg hatching
GO:0048568: embryonic organ development
GO:0019216: regulation of lipid metabolic process
GO:0007167: enzyme-linked receptor protein signaling pathway
R-HSA-452723: Transcriptional regulation of pluripotent stem cells
GO:1904062: regulation of cation transmembrane transport
GO:0048705: skeletal system morphogenesis
GO:0007610: behavior
hsa05205: Proteoglycans in cancer
hsa04550: Signaling pathways regulating pluripotency of stem cells
WP2853: Endoderm differentiation
GO:0097435: supramolecular fiber organization
GO:0034330: cell junction organization
GO:0035239: tube morphogenesis
GO:0006790: sulfur compound metabolic process
GO:0045913: positive regulation of carbohydrate metabolic process
GO:0007420: brain development
GO:0040008: regulation of growth
GO:0006066: alcohol metabolic process
GO:0040007: growth
B
ITPK1 MPI SLC25A12 GLDC TMEM106A CHST4 XYLT2 PDPR LDLR ALDH2 SLC45A3 GALNT18 NNT PLPP2 OGDHL NR3C1 KIT G6PD SORBS1 ELOVL5 IRS1 CPT1A ACER2 PHGDH G6PC3 PCK2 ACOT7 FGFR4 WDTC1 PTH1R ACADS MPC2 GAL SHPK MTOR DLAT PLCG2 ACSS2 PCSK9 ADCY1 PCCA MTHFD1 ACTN3 SORL1 SLC23A2 INSIG1 CHST2 APP AK2 SCARB1 LPIN1
iKO -DOX
iKO +DOX 2d
iKO +DOX 3d
C
Pyruvate
MPC2
Fatty acid
CPT1A
Pyruvate
ACADS
DLAT
Acetyl-CoA
Acyl-CoA
Citrate
Fatty acid synthesis
Acetyl-CoA
Mitochondrion
TCA cycle
ACSS2
Acetate
ALDH2
Acetaldehyde
Cytosol
D
Naïve NANOG iKO H9 hESCs
DOX - +2d +3d MW (kDa)
FLAG-Cas9 150
NANOG 37
H2BK5ac 15
H3ac 15
H3K27ac 15
H4ac 10
ACSS2 75
ALDH2 50
CPT1A 75
DLAT 75
MPC2 15
PDHB 37
H3 15
α-TUBULIN 50
Relative protein levels
H2BK5ac p=0.0009
H3K27ac p=0.0202 p=0.0187
H3ac p=0.0035 p=0.0057
H4ac p=0.0016 p=0.0037
ACSS2 p=0.0070
ALDH2 p=0.0209
CPT1A
DLAT p<0.0001 p=0.0041
MPC2 p=0.0004 p=0.0093
PDHB
NANOG p<0.0001 p=0.0020
iKO -DOX
iKO +DOX 2d
iKO +DOX 3d
E
Naïve SHhES8 hESCs in PXGL
NT iKO
DOX - +2d +3d - +2d +3d MW (kDa)
150
NANOG 37
H3ac 15
H4ac 10
ACSS2 75
ALDH2 50
CPT1A 75
DLAT 75
MPC2 15
PDHB 37
H3 15
α-TUBULIN 50
Relative protein levels
H3ac p=0.0110
H4ac p=0.0004
ACSS2 p=0.0223
ALDH2 p=0.0157
CPT1A p<0.0001
DLAT p=0.0086
MPC2 p=0.0186
PDHB
NANOG p<0.0001 p=0.0350
NT -DOX
NT +DOX 2d
NT +DOX 3d
iKO -DOX
iKO +DOX 2d
iKO +DOX 3d
F
Relative mRNA levels
p<0.0001 p=0.0041 p=0.0021 p<0.0001 p=0.0008 p<0.0001 p=0.0001
NANOG ACADS ACSS2 ALDH2 CPT1A MPC2 DLAT
NANOG iKD -DOX
NANOG iKD +DOX 3d
G
Naïve NANOG iKD SHhES8 hESCs
DOX - +3d MW(kDa)
HA 150
NANOG 37
H2BK5ac 15
H3K27ac 15
H3ac 15
H4ac 10
ACSS2 75
ALDH2 50
CPT1A 100
DLAT 75
MPC2 15
PDHB 37
H3 15
α-TUBULIN 50

◀ **Figure EV4. NANOG activates a set of genes involved in acetyl-CoA synthesis in naïve hESCs.**

(**A**) The GO analysis of 473 NANOG- occupied and *NANOG* depletion- upregulated (top) as well as 362 NANOG-occupied and *NANOG* depletion- downregulated (bottom) DEGs in naïve hESCs. The top 20 enriched terms are presented. (**B**) The heatmap depicting normalized mRNA levels of metabolism- related genes in enriched pathways shown in Fig. 4B, measured by our RNA-seq assays. The color represents Z-scores. Three biological replicates were used for each condition ($n = 3$).
(**C**) A schematic diagram of metabolic pathways of acetyl-CoA synthesis and putative NANOG targets associated with the pathway. (**D**) The representative western blot analysis result for protein levels of NANOG, histone acetylation modifications, and proteins related to acetyl-CoA synthesis in naïve H9 *NANOG* iKO hESCs cultured under the 5iLA condition, either untreated or treated with DOX for two or three days. Alpha-TUBULIN and histone 3 served as loading controls (left). The quantitative analysis results of relative protein levels are shown on the right side. Data are presented as mean ± SEM ($n = 3$). DLAT, Row 1, $p = 2.93 \times 10^{-7}$; NANOG, Row 1, $p = 2.60 \times 10^{-5}$.
(**E**) The representative western blot analysis result for levels of proteins indicated in naïve NT and *NANOG* iKO SHhES8 hESCs cultured under the PXGL condition, either untreated or treated with DOX for two or three days. Alpha-TUBULIN and histone 3 served as loading controls (left). The quantitative analysis results of relative protein levels are shown on the right side. Data are presented as mean ± SEM ($n = 3$). CPT1A, $p = 1.08 \times 10^{-5}$; NANOG, Row 1, $p = 1.06 \times 10^{-6}$. (**F**) RT-qPCR analysis results for relative mRNA levels of the indicated genes in naïve *NANOG* iKD SHhES8 hESCs, either untreated or treated with DOX for 3 days. Data are presented as mean ± SEM ($n = 3$). *NANOG*, $p = 9.39 \times 10^{-5}$; *ALDH2*, $p = 9.35 \times 10^{-6}$; *MPC2*, $p = 3.62 \times 10^{-5}$. (**G**) The representative western blot analysis result for levels of indicated proteins in naïve *NANOG* iKD SHhES8 hESCs, either untreated or treated with DOX for 3 days. dCas9 was tagged by HA. Alpha-TUBULIN and histone 3 served as loading controls. The unpaired two-tailed student's *t*-test was used for the statistical analysis in (**D–F**).

 © The Author(s)

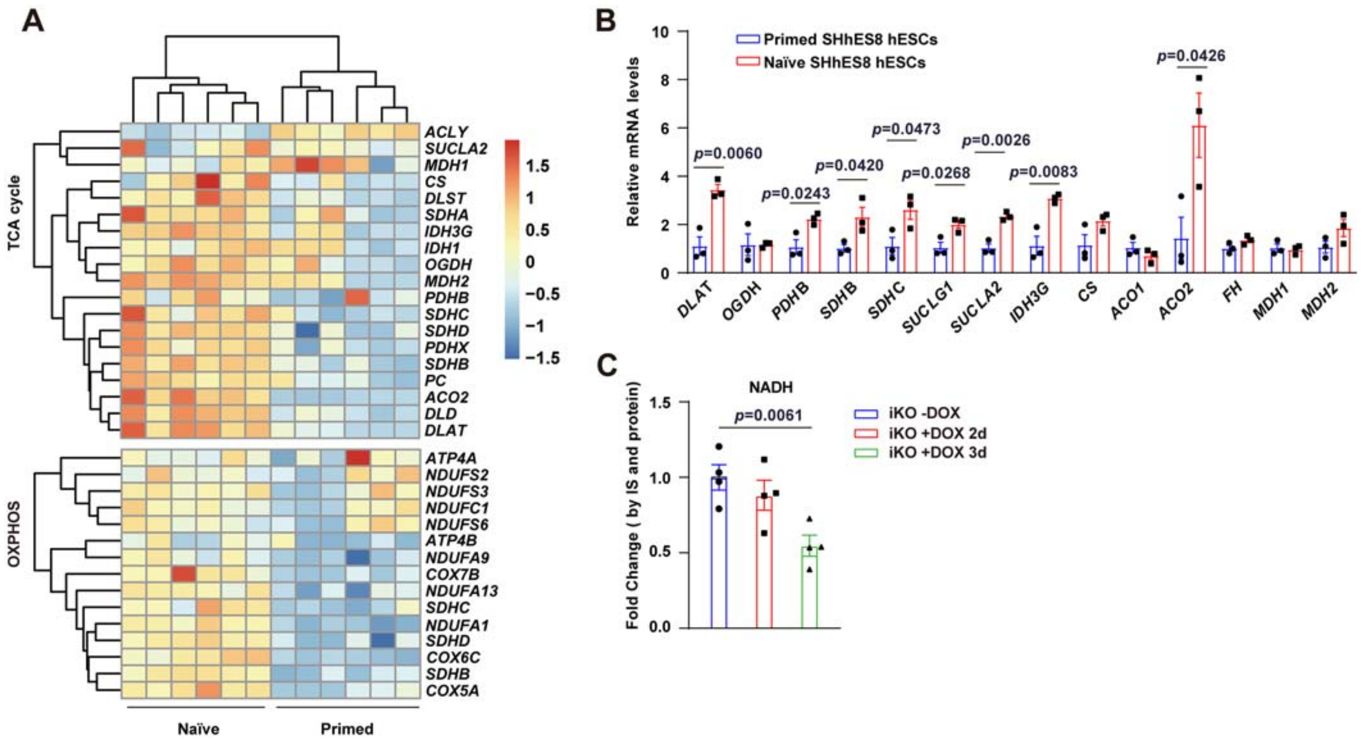

Figure EV5. Expression profiles of metabolism-associated genes and abundance of NADH.

(**A**) Heatmaps showing mRNA levels of genes related to the TCA cycle and OXPHOS in naïve and primed hESCs. Public RNA-seq datasets (GEO: GSE169678) and our RNA-seq datasets (SRA: PRJNA1258868) were used. The color represents Z-scores. Six biological replicates were used for each state of hESCs. (**B**) Results of RT-qPCR assays for the comparison in relative mRNA levels of TCA cycle- related genes between primed and naïve hESCs. Data are presented as mean ± SEM ($n = 3$). (**C**) Bar charts showing the relative abundance of NADH in naïve *NANOG* iKO SHhES8 hESCs, either untreated or treated with DOX for two or three days, as measured by the LC-MS analysis. Data are shown as mean ± SEM ($n = 4$). The unpaired two-tailed student's *t*-test was used for the statistical analysis in (**B**, **C**).

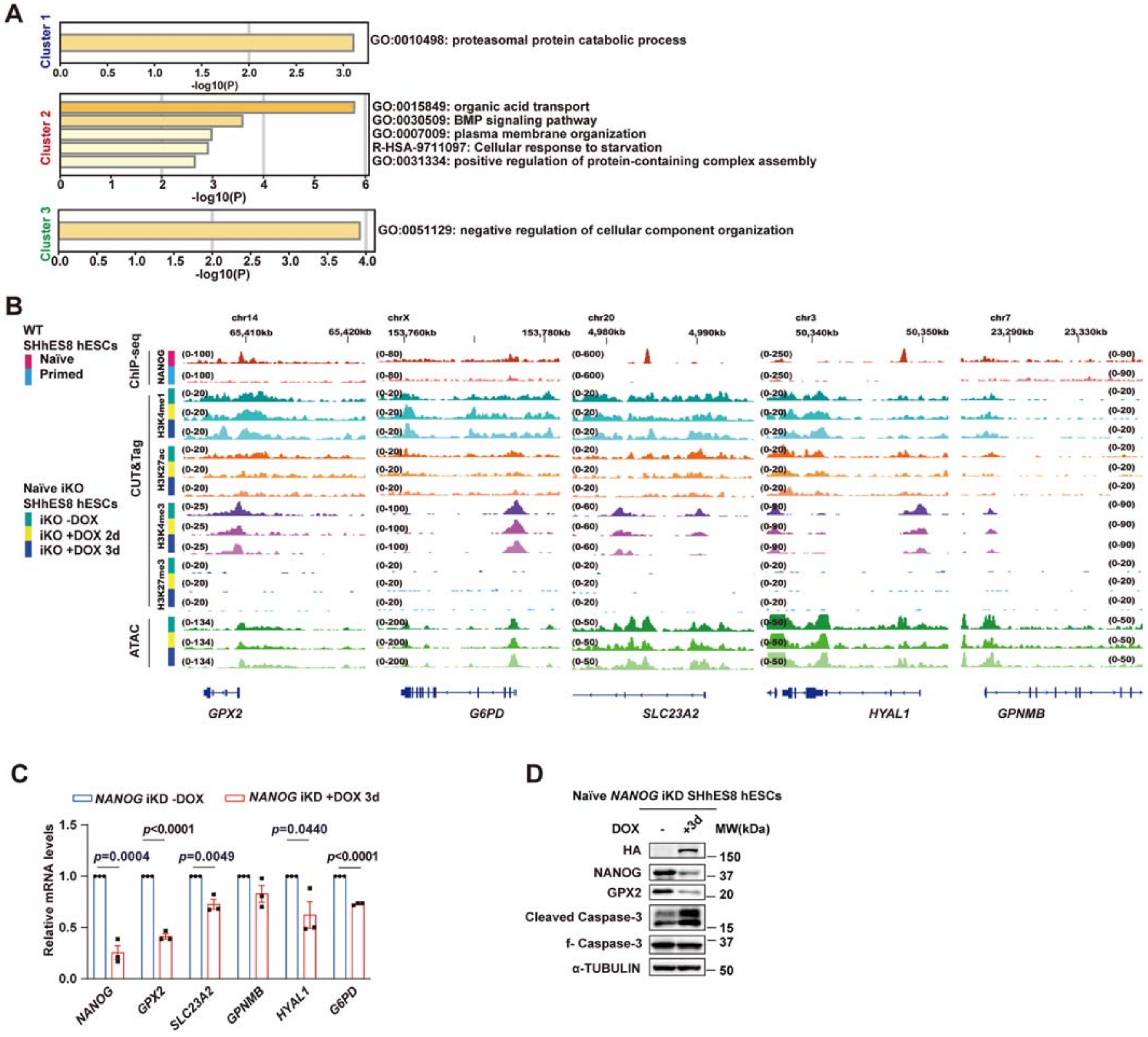

Figure EV6. NANOG directly regulates certain oxidative stress- related genes in naïve hESCs.

(**A**) Bar charts showing functional annotation of genes from the three clusters described in Fig. 6C. The hypergeometric test was used for statistical analysis. (**B**) Genome browser snapshots of NANOG occupancy at the vicinity of *GPX2*, *G6PD*, *SLC23A2*, *HYAL1*, and *GPNMB* in primed and naïve SHhES8 hESCs, as well as histone modifications and chromatin openness in naïve *NANOG* iKO SHhES8 hESCs, either untreated or treated with DOX for two or three days. (**C**) RT-qPCR analysis results for relative mRNA levels of indicated genes in naïve *NANOG* iKD SHhES8 hESCs, either untreated or treated with DOX for three days. Data are presented as mean ± SEM ($n = 3$). *GPX2*, $p = 2.48 \times 10^{-5}$; *G6PD*, $p = 2.31 \times 10^{-6}$. The unpaired two-tailed student's *t*-test was used for statistical analysis. (**D**) The representative western blot analysis result for levels of indicated proteins in naïve *NANOG* iKD SHhES8 hESCs, either untreated or treated with DOX for three days. dCas9 was tagged by HA. Alpha-TUBULIN served as a loading control.

 © The Author(s)

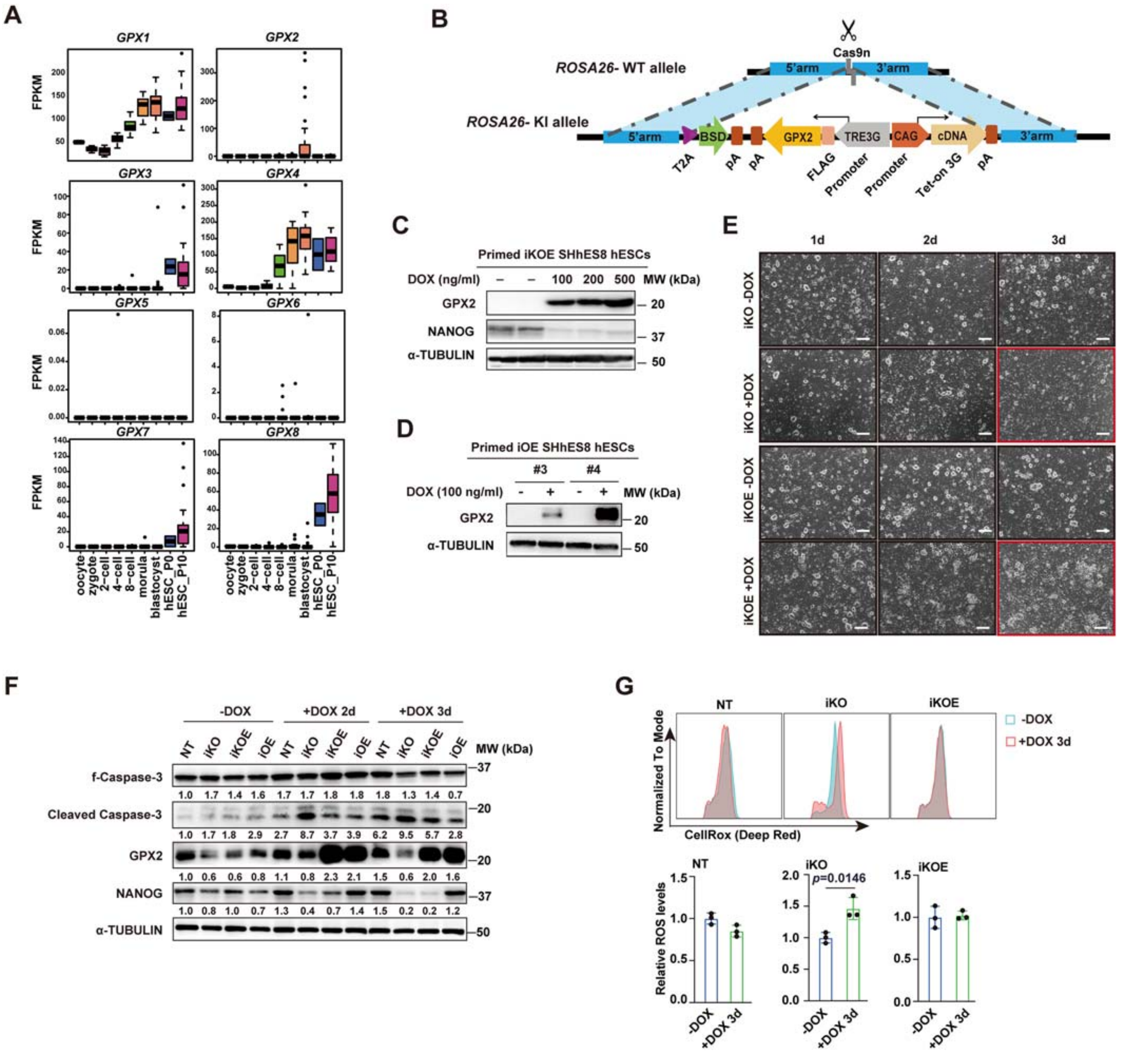

Figure EV7. ***GPX2*** **expression profiles and its rescue effect on** ***NANOG*** **depletion- induced oxidative stress and cell death in naïve hESCs.**

(**A**) The expression profile of glutathione peroxidase family members during human early embryonic development and in cultured hESCs. Data were obtained from the published dataset (GEO: GSE36552). (**B**) The schematic diagram showing the insertion of the cassette for DOX inducible overexpression of *GPX2* into the ROSA26 locus of hESCs. (**C**, **D**) The representative western blot analysis results of GPX2 and NANOG protein levels in primed iKOE (**C**) and GPX2 in iOE (**D**) SHhES8 hESCs, either untreated or treated with DOX for three days. Alpha-TUBULIN served as a loading control. (**E**) Phase contrast images of naïve *NANOG* iKO and iKOE SHhES8 hESCs, either untreated or treated with DOX. Scale bar, 200 μm. (**F**) The representative western blot analysis result for levels of indicated proteins in naïve NT, iKO, iKOE, and iOE SHhES8 hESCs, either untreated or treated with DOX for two or three days. Alpha-TUBULIN served as a loading control. The number under each row indicates the relative protein abundance measured by ImageJ. (**G**) The representative ROS peak graph calculated by the CellROX staining combined with flow cytometric analysis in naïve NT, iKO, and iKOE hESCs cultured in the PXGL medium, treated without or with DOX (top). The quantitative analysis of relative ROS levels from three independent experiments are shown at the bottom. Data are shown as mean ± SD ($n = 3$). The unpaired two-tailed Student's t-test was used for statistical analysis.
